# Dynamics of severity-associated immune remodeling by granulocytes and macrophages in acute lung injury

**DOI:** 10.1016/j.isci.2026.115816

**Published:** 2026-04-21

**Authors:** Wanqin Zeng, Caijin Wang, Chengjian Cao, Chunlai Nie, Yingjun Fan, Yi Zhang, Zhongshan He

**Affiliations:** 1 State Key Laboratory of Biotherapy and Cancer Center, Research Unit of Gene and Immunotherapy, Chinese Academy of Medical Sciences, Collaborative Innovation Center of Biotherapy, West China Hospital, Sichuan University, Chengdu, Sichuan 610041, China; 2 Sichuan Institute of Industry and Information Technology, Chengdu, Sichuan 610041, China; 3 Zigong Academy of Medical Sciences, Zigong First People’s Hospital, Zigong, Sichuan 643000, China; 4 Department of Ultrasound, Department of Neurosurgery, The First Affiliated Hospital of Chengdu Medical College, Chengdu, Sichuan 610500, China; 5 Departments of Molecular Physiology and Biological Physics and Department of Biomedical Engineering, University of Virginia, Charlottesville, VA 22903, USA

**Keywords:** biological sciences

## Abstract

Acute lung injury (ALI) is driven by dysregulated alveolar immune responses. While granulocytes and macrophages are critical effectors, their coordinated molecular reprogramming and severity-associated crosstalk shape immune remodeling during disease progression. Here, we performed single-cell RNA sequencing (scRNA-seq) of bronchoalveolar lavage fluid from patients with intermediate- and late-severity ALI to map immune microenvironment remodeling. We observed pronounced granulocyte expansion coupled with macrophage depletion. Granulocytes exhibit distinct transcriptional states along a continuum from acute migratory states toward pro-inflammatory, metabolically reprogrammed subsets, dominated by TNF and MAPK signaling. Simultaneously, macrophages shift toward inflammatory M1-like phenotypes, characterized by distinct metabolic reprogramming and reduced oxidative phosphorylation. Furthermore, we infer a granulocyte-centric inflammatory network mediated through TNF, IFN, and RESISTIN pathways, suggesting a feedforward inflammatory loop. Collectively, this study elucidates the transcriptional and metabolic reprogramming associated with escalating ALI severity, providing a framework for severity-adapted therapeutic interventions to restore pulmonary homeostasis.

## Highlights

• scRNA-seq maps severity-stratified immune remodeling in human ALI
• Granulocytes expand and show metabolic rewiring in high-severity ALI
• TNF/RESISTIN-linked myeloid signaling is enhanced in high-severity ALI
• p38 MAPK signaling and OXPHOS impairment align with severe ALI hyperinflammation

## Graphical abstract

**Figure:**
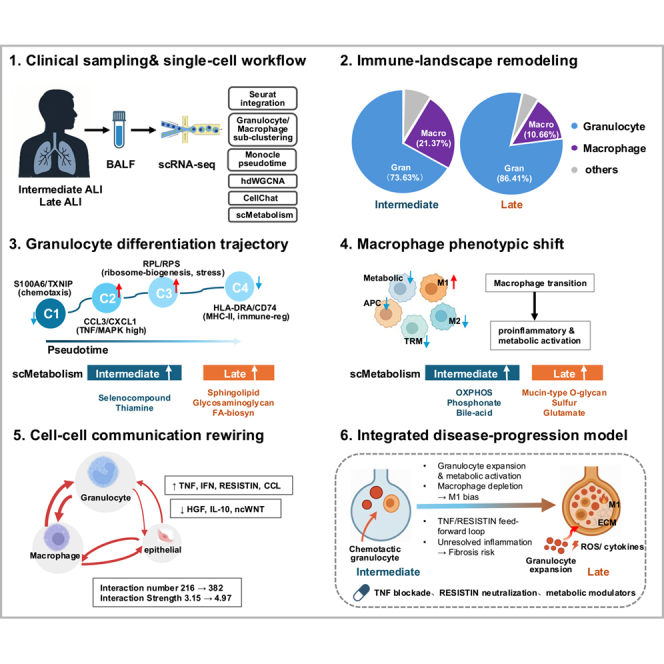

## Introduction

Acute lung injury (ALI) and its severe form, acute respiratory distress syndrome (ARDS), are life-threatening conditions characterized by diffuse alveolar damage and excessive inflammation, leading to substantial morbidity and mortality.^1^^,^^2^^,^^3^ Although advancements in supportive care have improved overall management, current therapies remain largely non-specific, lacking the precision to modulate immune responses without compromising host defense.^4^^,^^5^^,^^6^ Therefore, a deeper understanding of the pathogenic mechanisms and cellular interactions across different stages of ALI in patients is critical for developing precise and effective therapeutic strategies.

The progression of ALI arises from persistent immune dysregulation, driven by unbalanced inflammatory responses and defective resolution mechanisms, ultimately leading to maladaptive tissue repair and impaired lung recovery.^7^^,^^8^ Innate immune cells, particularly granulocytes and monocyte-derived macrophages, play critical roles in this process.^9^ Granulocytes initiate acute inflammation by releasing reactive oxygen species and proteases and by recruiting additional immune cells, thereby contributing both to host defense and to alveolar damage. Meanwhile, macrophages act as central regulators of the immune microenvironment through the clearance of apoptotic cells and pathogens, the production of cytokines, and the orchestration of inflammation resolution and tissue repair.^10^^,^^11^^,^^12^^,^^13^ However, most classical and contemporary ALI/ARDS studies—both experimental and clinical—have characterized neutrophils and macrophages either in bulk, at a single time point, or in isolation, focusing primarily on their individual contributions to lung injury.^14^^,^^15^ Consequently, how the transcriptional states, metabolic programs, and interaction networks of these two lineages co-evolve across clinically distinct stages of human ALI remains largely unknown. This critical knowledge gap limits our understanding of why inflammation fails to resolve in late-severity disease and hinders the development of stage-adapted therapeutic strategies.

To address this knowledge gap, we systematically investigated the stage-specific roles and interplay of granulocytes and macrophages in ALI. Here, we performed single-cell RNA sequencing (scRNA-seq) on bronchoalveolar lavage fluid (BALF) from patients with intermediate- and late-severity disease to delineate immune microenvironmental evolution. To our knowledge, no prior study has delineated a stage-specific, granulocyte-centered signaling circuit in human ALI using integrative single-cell approaches. Unlike broad single-cell atlases that emphasize global cell-type heterogeneity,^16^^,^^17^^,^^18^ our study explicitly reconstructs the coordinated reprogramming of granulocytes and macrophages. Specifically, we mapped their differentiation trajectories, metabolic shifts, and intercellular communication networks. Our analyses reveal that late-severity ALI is not merely a prolongation of early inflammation but represents a distinct state characterized by a granulocyte-centric inflammatory network. We demonstrate that granulocytes persistently expand and emerge as major signaling hubs, broadcasting TNF, IFN, and RESISTIN signals that drive sustained inflammation. Simultaneously, macrophages undergo a marked decline in number but adopt a distinct M1-like phenotype characterized by impaired oxidative phosphorylation.

These mechanistic insights provide a systems-level view of the non-resolving inflammatory state. By clarifying the precise temporal dynamics and functional shifts of granulocytes and macrophages, our findings support a conceptual shift toward stage-adapted immunomodulation—targeting granulocyte-centric circuits in late disease while preserving early host defense—thereby providing a framework for precision management in ALI.

## Results

### Overview of the severity-stratified single-cell experimental design and integrated analytical workflow

**Figure 1:**
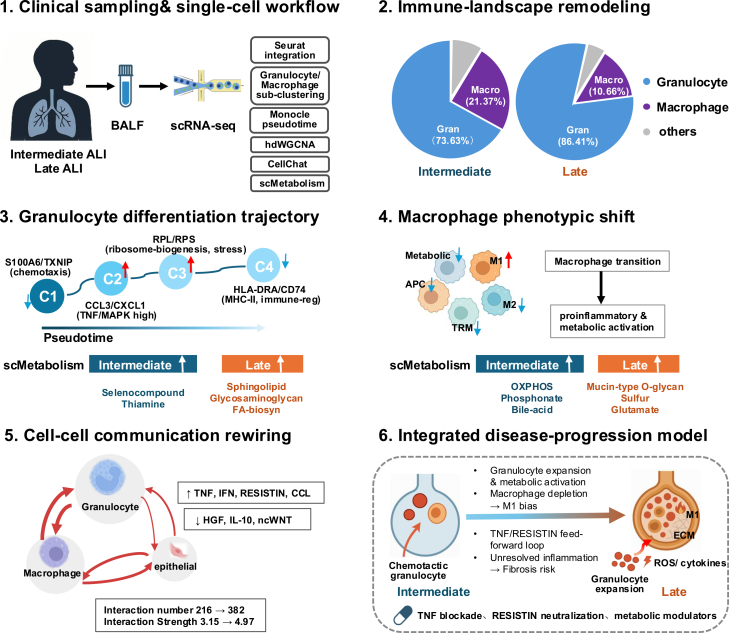
Schematic summary of dynamic immune reprogramming in progressive acute lung injury (1) Clinical sampling and single-cell workflow illustrate the collection of bronchoalveolar lavage fluid (BALF) from patients with intermediate and late-severity ALI, followed by single-cell RNA sequencing (scRNA-seq). Computational analyses included Seurat integration, granulocyte/macrophage sub-clustering, pseudotime trajectory analysis (Monocle), co-expression network analysis (hdWGCNA), cell-cell communication analysis (CellChat), and metabolic profiling (scMetabolism). (2) Immune-landscape remodeling characterized by increased granulocyte (Gran) proportions and decreased macrophage (Macro) populations from intermediate to late ALI stages. (3) Granulocyte differentiation trajectory analyzed via pseudotime, depicting sequential cluster transitions (C1 to C4) from infiltrating (S100A6/TXNIP), inflammatory (CCL3/CXCL1), ribosomal/stress (RPL/RPS) to immunoregulatory (HLA-DRA/CD74) states, accompanied by metabolic shifts from early-severity selenocompound and thiamine metabolism toward late-severity sphingolipid, glycosaminoglycan, and fatty acid biosynthesis (FA-biosyn). (4) Macrophage phenotypic shift from intermediate multifunctional subtypes (Metabolic, APC, TRM, and M2) toward a pro-inflammatory, M1-dominated phenotype in late-severity disease. This transition is underpinned by metabolic changes from oxidative phosphorylation (OXPHOS), phosphonate, and bile-acid metabolism to increased mucin-type O-glycan, sulfur, and glutamate metabolism. (5) Cell-cell communication rewiring, highlighting intensified granulocyte-driven inflammatory signaling networks involving TNF, IFN, RESISTIN, and CCL pathways, coupled with decreased reparative signaling (HGF, IL-10, ncWNT) from intermediate to late-severity ALI. Interaction numbers and strengths markedly increase in late-severity disease, indicating amplified cellular crosstalk. (6) Integrated disease-progression model summarizes granulocyte expansion, macrophage polarization toward M1, sustained inflammatory signaling via TNF/RESISTIN feedforward loops, and resultant unresolved inflammation and fibrosis risk. Proposed therapeutic intervention points include TNF blockade, RESISTIN neutralization, and metabolic modulators. Icons in (1), (4), and (5) were created with BioRender.com.

Figure 1 presents a schematic illustration of the immune remodeling and intercellular rewiring observed during severity-associated ALI. Initially, we outline the clinical sampling and scRNA-seq workflow, highlighting the isolation and enrichment of immune cells from BALF and subsequent computational analyses. The severity-associated immune landscape remodeling is demonstrated, showing increased granulocyte proportions and decreased macrophages from intermediate-to late-severity strata. Granulocyte differentiation trajectories identified by pseudotime analysis illustrate shifts through chemotactic, inflammatory, and immunoregulatory states, alongside associated metabolic reprogramming. Concurrently, macrophages shift from a multifunctional, homeostatic profile toward a pro-inflammatory, M1-dominated phenotype, underscored by metabolic shifts including increased mucin-type O-glycan, sulfur, and glutamate metabolism. Enhanced intercellular communication, especially between granulocytes, macrophages, and epithelial cells, is emphasized through augmented TNF, IFN, and RESISTIN signaling, suggesting a self-perpetuating inflammatory loop. Finally, an integrated severity-associated model summarizes these immune dynamics, emphasizing granulocyte expansion, macrophage polarization toward a proinflammatory phenotype, sustained inflammatory signaling, and increased fibrosis risk, while suggesting potential therapeutic targets such as TNF blockade, RESISTIN neutralization, and metabolic modulators.

### Single-cell profiling reveals marked granulocyte expansion and macrophage depletion as hallmarks of late-severity ALI

**Figure 2:**
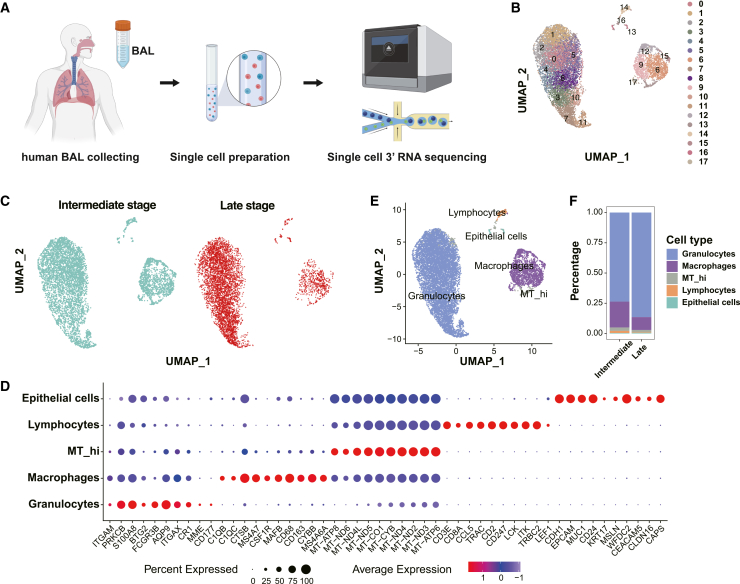
Single-cell transcriptomic profiling reveals immune landscape remodeling across progressive stages of lung injury (A) Schematic overview of the experimental workflow. Bronchoalveolar lavage fluid (BALF) samples from patients with intermediate and late-severity lung injury were enzymatically dissociated into single-cell suspensions and subjected to single-cell 3′ RNA sequencing using the 10× Genomics platform. (B) UMAP plot of 18 transcriptionally distinct cell clusters identified from all high-quality cells, revealing prominent cellular heterogeneity across the dataset. (C) UMAP projections stratified by clinical stage. Cells from intermediate (blue) and late-severity (red) samples form partially overlapping but distinct distributions, indicating progressive shifts in cellular composition. (D) Dot plot displays the average expression (color intensity) and detection rate (dot size) of canonical marker genes across five major cell types. Each cell population exhibits distinct marker gene signatures (e.g., CXCL8, IL1B in granulocytes; FCGR3A, TREM2 in macrophages), enabling robust annotation. (E) UMAP plot colored by annotated cell types, highlighting five major compartments: granulocytes, macrophages, epithelial cells, lymphocytes, and MT_hi cells. Granulocytes dominate the transcriptional space, while other lineages form discrete clusters. (F) Bar plot shows the relative frequency of each cell type across disease stages. A marked increase in granulocytes and a concomitant decline in macrophages are observed in late-severity samples, reflecting a shift toward a granulocyte-dominated inflammatory milieu. Icon in (A) was created with BioRender.com.

To systematically elucidate the severity-associated immune alterations underlying the progression of ALI, we performed scRNA-seq on BALF samples obtained from patients stratified by intermediate and late severity at sampling (Figure 2A). Following enzymatic dissociation and meticulous library preparation, high-quality single-cell transcriptomes were generated, enabling comprehensive profiling of cellular heterogeneity within BALF cells.

After rigorous quality control and filtering procedures (Figure S1), a total of 10,504 cells (intermediate: 5,551 cells; late: 4,953 cells) were integrated, clustered, and visualized using uniform manifold approximation and projection (UMAP) via the Seurat R package. Through unsupervised clustering, 18 transcriptionally distinct clusters were identified across the integrated dataset (Figure 2B), reflecting the considerable immune diversity present within the alveolar niche. Subsequently, severity stratification of these clusters revealed distinct severity-associated compositional shifts in cell populations (Figure 2C).

Next, major cell types were annotated using established canonical markers.^19^ These included granulocytes (S100A8, FCGR3B, ITGAM),^20^ macrophages (CTSB, CD68, MS4A7),^21^ epithelial cells (EPCAM, KRT17, CDH1),^22^ lymphocytes (CD3E, TRAC, LCK), and metabolically active MT_hi cells (MT-ND4L, MT-CO1, MT-CYB) (Figures 2D, 2E, and S2). As anticipated, granulocytes and macrophages constituted dominant compartments in both stages, consistent with established paradigms of granulocytic infiltration and monocyte-derived immune activation in ALI.^23^

Importantly, quantitative comparisons revealed a substantial increase in granulocyte abundance during the late-severity stratum, suggesting persistent granulocyte recruitment and unresolved inflammation. Conversely, macrophage proportions were reduced in the late stage compared to the intermediate phase (Figure 2F), indicating a possible decline in mononuclear phagocyte activity or altered cellular recruitment dynamics. This divergence from classical inflammation-resolution trajectories suggests sustained innate immune activation and highlights a potential mechanism contributing to tissue damage and worsening severity in ALI.

Collectively, these findings define a severity-associated immune landscape in ALI, marked by late-severity granulocyte predominance and amplified inflammatory crosstalk, alongside reduced macrophage abundance but enhanced pro-inflammatory polarization. This remodeling may sustain non-resolving inflammation and promote fibrotic remodeling, offering a foundation for investigating severity-dependent cell-state transitions and intercellular signaling.

### Severity-associated transcriptional reprogramming drives inflammatory amplification in granulocytes and functional shifts in macrophages

**Figure 3:**
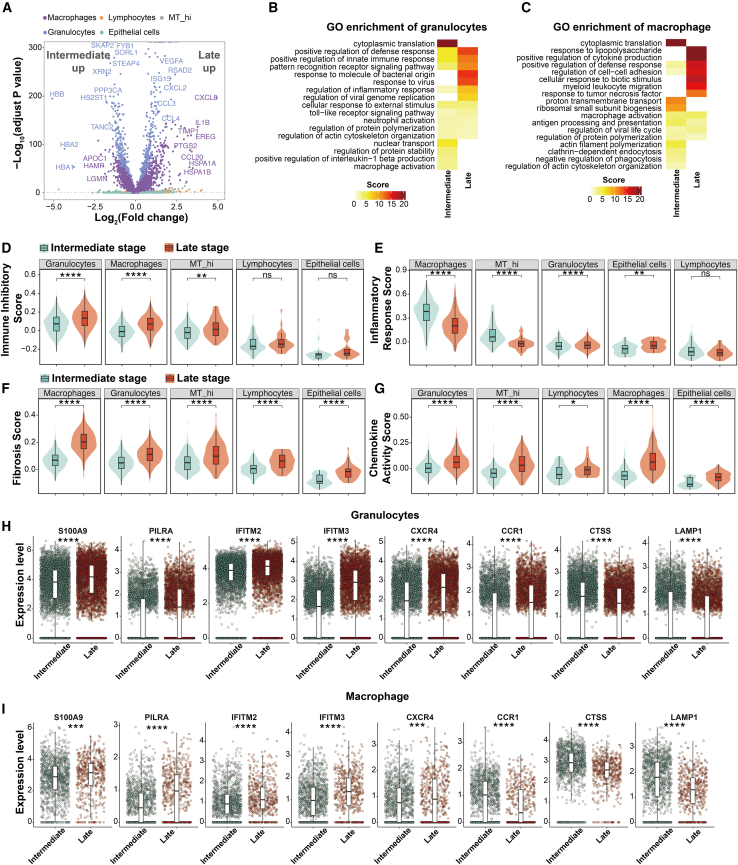
Divergent transcriptional dynamics of granulocytes and macrophages during intermediate and late-severity lung injury (A) Volcano plot shows differentially expressed genes between intermediate and late stages across major immune cell types. Genes enriched in granulocytes (blue) and macrophages (purple) are prominently labeled, illustrating distinct effector programs in each lineage. (B and C), Gene Ontology enrichment analysis of differentially expressed genes in granulocytes (B) and macrophages (C) at intermediate and late stages. Granulocytes exhibit enrichment for viral and bacterial defense pathways and innate immune activation in the late stage, while macrophages demonstrate enhanced cytokine signaling and migration programs. (D) Violin plots show immune inhibitory scores across five major cell populations. Granulocytes and macrophages exhibit elevated suppression signatures at the late stage. (E) Inflammatory response scores increase in granulocytes but decrease in macrophages during progression, highlighting a shift in the dominant inflammatory compartment. (F) Fibrosis scores rise in granulocytes, macrophages, and MT_hi cells in the late stage, implicating their involvement in tissue remodeling. (G) Chemokine activity scores are elevated in granulocytes and macrophages, indicating increased signaling capacity for leukocyte recruitment. (H and I), Boxplots display gene expression levels of effector genes across stages in granulocytes (H) and macrophages (I). Granulocytes upregulate S100A9, PILRA, IFITM2/3, CXCR4, and CCR1, while CTSS and LAMP1 are downregulated. Macrophages display similar upregulation of interferon-response genes, but show reduced expression of CCR1, CTSS, and LAMP1 in the late stage. Statistical significance was assessed using the Wilcoxon rank-sum test; *p* < 0.05 (∗), <0.01 (∗∗), <0.001 (∗∗∗), and <0.0001 (∗∗∗∗); ns, not significant.

Building on the previously defined cellular landscape, we next investigated the molecular alterations that accompany disease progression from the intermediate to late severity strata of ALI. Cell-level differential gene expression analysis revealed substantial transcriptional reprogramming across immune cell populations, with granulocytes and macrophages exhibiting the most pronounced shifts (Figure 3A). In particular, late-severity granulocytes displayed higher expression of ISG15, CCL3, VEGFA, CCL4, CXCL2, and RSAD2, reflecting enhanced interferon signaling, angiogenesis-associated remodeling, and chemokine-mediated leukocyte recruitment. In parallel, macrophages from late-severity samples showed higher expression of key inflammatory and stress response genes, including CXCL8, IL1B, TIMP1, CCL20, HSPA1A, HSPA1B, EREG, and PTGS2, indicating the activation of cytokine production, cellular stress pathways, and matrix remodeling processes.

To further dissect these lineage-specific transcriptional changes, we conducted Gene Ontology (GO) enrichment analysis. Late-severity granulocytes were enriched for defense response activation, innate immune signaling, pattern recognition receptor pathways, and responses to bacterial and viral components (Figure 3B), reinforcing their role in pathogen sensing and effector responses. By contrast, intermediate-severity granulocytes were enriched for cytoplasmic translation, protein stabilization, and macrophage activation pathways, suggesting a more regulated inflammatory profile at earlier stages. Similarly, macrophages in late-severity samples showed enrichment for lipopolysaccharide response, cytokine production, cell adhesion, and myeloid cell migration (Figure 3C), while intermediate-severity macrophages were primarily associated with ribosomal biogenesis, actin cytoskeleton regulation, and phagocytic activity—indicative of a transitional, homeostatic state.

Next, we performed the quantitative assessment of phenotypic dynamics using curated gene module scoring for immune inhibition, inflammatory responses, fibrosis, and chemotactic activity (Figures 3D–3G). Notably, both granulocytes and macrophages in the late severity stratum exhibited elevated immune inhibitory scores (Figure 3D), suggesting concurrent engagement of checkpoint-associated programs and inflammatory signaling. Interestingly, inflammatory response scores diverged by cell type—granulocytes demonstrated an increase, while macrophages showed a decline (Figure 3E), indicating a cell-type shift toward granulocyte-dominant inflammation in advanced lesions. In addition, fibrosis-related scores were elevated in granulocytes, macrophages, and metabolically active MT_hi cells (Figure 3F), implicating these populations in fibrotic remodeling. Chemokine activity scores also rose across granulocytes and macrophages in late-severity samples (Figure 3G), reflecting sustained inflammatory recruitment and signaling.

To validate these patterns, we examined the expression of key effector genes. In granulocytes, genes associated with interferon signaling and chemotaxis, including S100A9, PILRA, IFITM2, IFITM3, CXCR4, and CCR1, were upregulated in late-severity samples. Conversely, genes involved in lysosomal activity, such as CTSS and LAMP1, were downregulated (Figure 3H), pointing to a functional transition away from proteolysis. Similarly, macrophages mirrored this pattern, with the elevated expression of S100A9, PILRA, IFITM2, IFITM3, and CXCR4, and downregulation of CCR1, CTSS, and LAMP1 (Figure 3I), further supporting their involvement in proinflammatory activation and reprogrammed degradation pathways. Next, we isolated primary CD45^+^CD66b^+^ granulocytes and CD45^+^CD68^+^ macrophages from BALF of clinically defined patients with intermediate- and late-severity ALI. Consistent with the scRNA-seq findings, our RT-qPCR analysis demonstrated that late-severity cells exhibited a consistent pattern of increased expression in key inflammatory markers and downregulation in lysosomal genes, thereby providing robust validation of the severity-associated transcriptional reprogramming inferred from the single-cell atlas (Figures S3A and S3B).

Taken together, these findings define severity-associated, cell-type-specific transcriptional programs linked to worsening ALI severity. Granulocytes emerge as dominant contributors to late-severity inflammation, while macrophages exhibit transcriptional signatures associated with immunomodulation and fibrotic transition. These dynamic shifts in inflammatory and metabolic programming provide critical mechanistic insight and identify potential targets for therapeutic intervention in severe lung injury.

### Granulocyte trajectory analysis identifies a progression from acute migratory to hyperinflammatory and metabolically active states

**Figure 4:**
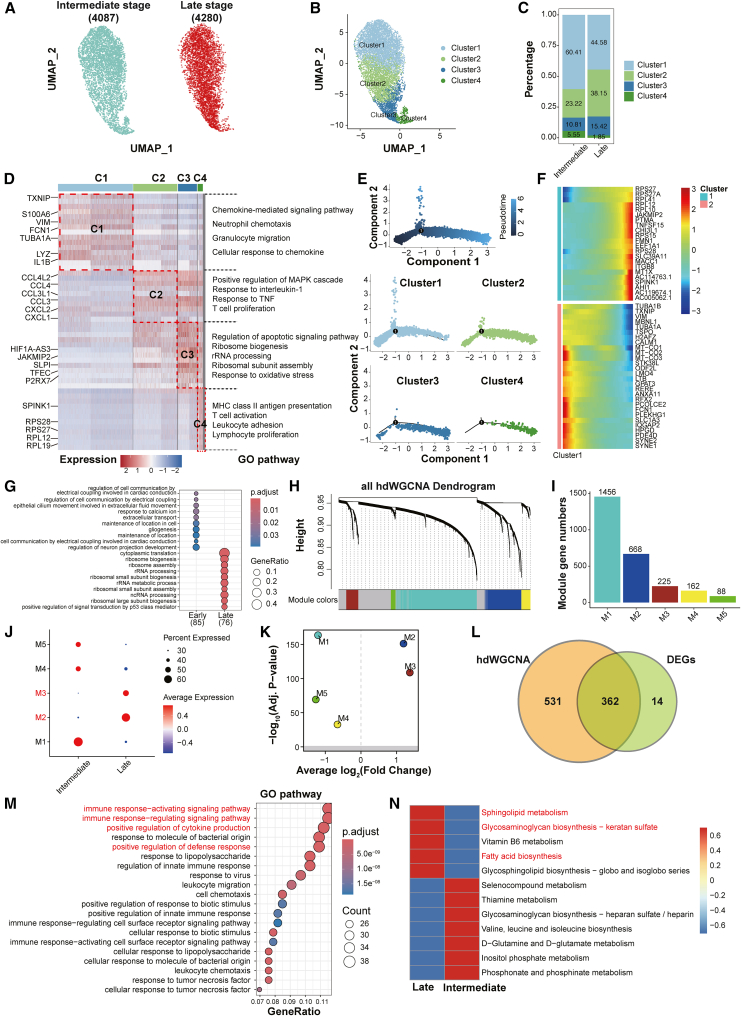
Functional heterogeneity and transcriptional dynamics of granulocyte subpopulations during lung injury (A) UMAP visualization of granulocytes from intermediate (left, *n* = 4,087) and late-severity (right, *n* = 4,280) lung injury samples, colored by disease stage. (B) Unsupervised clustering of granulocytes identified four transcriptionally distinct subpopulations (Clusters 1–4), each associated with unique gene expression programs. (C) Stacked bar plot shows the proportion of each granulocyte cluster across intermediate and late stages. Cluster 1 dominated in the intermediate stage (60.41%), whereas clusters 2 and 3 expanded in the late stage (38.15% and 15.42%, respectively), and cluster 4 decreased (1.85%). (D) Heatmap of representative marker genes across clusters, grouped by pathway annotations. Cluster 1 was enriched for chemokine signaling and granulocyte chemotaxis; cluster 2 for MAPK signaling, TNF response, and inflammation amplification; cluster 3 for ribosomal biogenesis, oxidative stress response, and apoptotic priming; and cluster 4 for antigen presentation and T cell activation. (E) Pseudotime trajectory analysis revealed a progression from clusters 1 to Cluster 4, with transcriptional maturation aligned with disease advancement. (F) Heatmaps of early (top) and late (bottom) pseudotime high-expression genes. (G) Gene Ontology (GO) enrichment analysis of pseudotime stage-specific genes revealed early-severity enrichment in epithelial movement and neuron projection regulation, and late-severity enrichment in ribosomal assembly and p53 signaling. (H) Hierarchical clustering dendrogram generated by hdWGCNA of granulocytes, identifying 5 distinct co-expression modules based on transcriptional similarities. (I) Bar plot shows gene counts per hdWGCNA module. (J) Dot plot shows module-specific gene expression levels and proportions across stages. (K) Volcano plot shows stage-specific differential expression of modules; M2 and M3 were upregulated in late-severity samples. (L) Venn diagram shows overlap between late-severity upregulated genes and M2/M3 modules (*n* = 362 shared genes). (M) GO enrichment analysis of the 362 intersecting genes revealed the activation of innate immune and cytokine response pathways. (N) KEGG pathway analysis revealed stage-specific metabolic rewiring: sphingolipid and glycosaminoglycan biosynthesis pathways were enriched in the late stage, whereas amino acid and inositol phosphate metabolism predominated in the intermediate stage.

Given the substantial expansion of granulocytes observed in late-severity lung injury (Figure 2F), we next sought to delineate their transcriptional dynamics and potential lineage transitions. To this end, we performed unsupervised reclustering of granulocytes, which identified four transcriptionally distinct subpopulations (clusters 1–4) (Figures 4A and 4B). Quantitative analysis of cluster distribution revealed stage-specific compositional shifts: Cluster 1 dominated in the intermediate severity stratum (60.41%) but decreased to 44.58% in the late stage, while Clusters 2 and 3 exhibited increases (23.22%–38.15% and 10.81%–15.42%, respectively). By contrast, Cluster 4 was markedly depleted in late-severity samples (5.55%–1.85%) (Figure 4C). These results suggest a dynamic continuum of granulocyte activation and differentiation with increasing ALI severity.

To characterize the functional heterogeneity of these granulocyte subsets, we next analyzed cluster-specific gene expression profiles and pathway enrichment patterns (Figure 4D). Cluster 1, predominant in intermediate-severity samples, showed strong expression of genes associated with leukocyte motility and infiltration, such as S100A6^24^ and TXNIP,^25^ and was enriched for acute granulocyte migration and chemokine signaling, representing an early infiltrating phenotype. In contrast, Cluster 2, which expanded in the late stage, displayed elevated chemokine ligand production (e.g., CCL3 and CXCL1) and activation of MAPK and TNF pathways, indicative of an amplified inflammatory state that orchestrates secondary leukocyte recruitment and tissue retention. Cluster 3 was marked by the upregulation of genes involved in ribosome biogenesis and oxidative stress, consistent with metabolic reprogramming in response to sustained stimulation. Meanwhile, Cluster 4, which diminished in late-severity samples, was enriched in MHC-II-related and T cell-activating pathways, suggesting a potential immunoregulatory function. Together, these profiles illustrate a pseudotime-inferred shift from early migratory to inflammatory and regulatory granulocyte states.

Subsequently, to map potential differentiation trajectories among granulocyte subsets, we employed pseudotemporal ordering using Monocle.^26^ The inferred trajectory suggested a pseudotime ordering from Cluster 1 to Cluster 4, proceeding through intermediate states represented by Clusters 2 and 3 (Figure 4E). This sequential pattern is consistent with granulocytes shifting from acute migratory and proinflammatory phenotypes toward more biosynthetic and immunoregulatory roles. Supporting this trajectory, pseudotime gene ordering showed that early-phase genes were enriched in pathways related to cell-cell communication, cilium movement, and calcium signaling, while late-phase genes were associated with ribosome assembly, rRNA processing, and p53 signaling (Figures 4F and 4G), suggesting transcriptional reprogramming aligned with stress adaptation and protein synthesis.

To further uncover coordinated gene programs underlying these transitions, we performed high-dimensional weighted gene co-expression network analysis (hdWGCNA),^27^ which identified five major gene modules (M1-M5) (Figures 4H and 4I). Among these, modules M2 and M3 were enriched in late-severity granulocytes (Figures 4J and 4K). Venn diagram analysis demonstrated a strong overlap between M2/M3 modules and late-severity upregulated genes (*n* = 362 genes), confirming the robustness and relevance of these signatures (Figure 4L). GO enrichment analysis of these intersecting genes highlighted critical immune activation pathways, including cytokine production, response to LPS and viral stimuli, and activation of innate immune signaling cascades (Figure 4M).

Furthermore, we examined the metabolic adaptations accompanying granulocyte reprogramming. Metabolic enrichment analysis revealed that late-severity granulocytes were enriched in biosynthetic pathways such as sphingolipid metabolism, glycosaminoglycan biosynthesis, vitamin B6 metabolism, and fatty acid biosynthesis (Figure 4N). These pathways are linked to membrane remodeling, secretory granule formation, and lipid-driven inflammation, suggesting an effector-like state in granulocytes. In contrast, intermediate-severity granulocytes displayed higher activity in selenocompound metabolism, thiamine metabolism, inositol phosphate turnover, and amino acid biosynthesis (e.g., valine and glutamate), reflecting metabolic priming for redox balance and early-severity immune activation.

Collectively, these integrated analyses define a transcriptional and metabolic continuum of granulocyte differentiation during lung injury. This continuum spans from early infiltrating and inflammatory states in the intermediate stage to biosynthetic and immunoregulatory phenotypes in the late stage, offering mechanistic insights into granulocyte adaptation during persistent alveolar inflammation.

### Macrophage subsets undergo a shift toward M1-like polarization associated with reduced oxidative phosphorylation and increased mucin-type O-glycan biosynthesis

**Figure 5:**
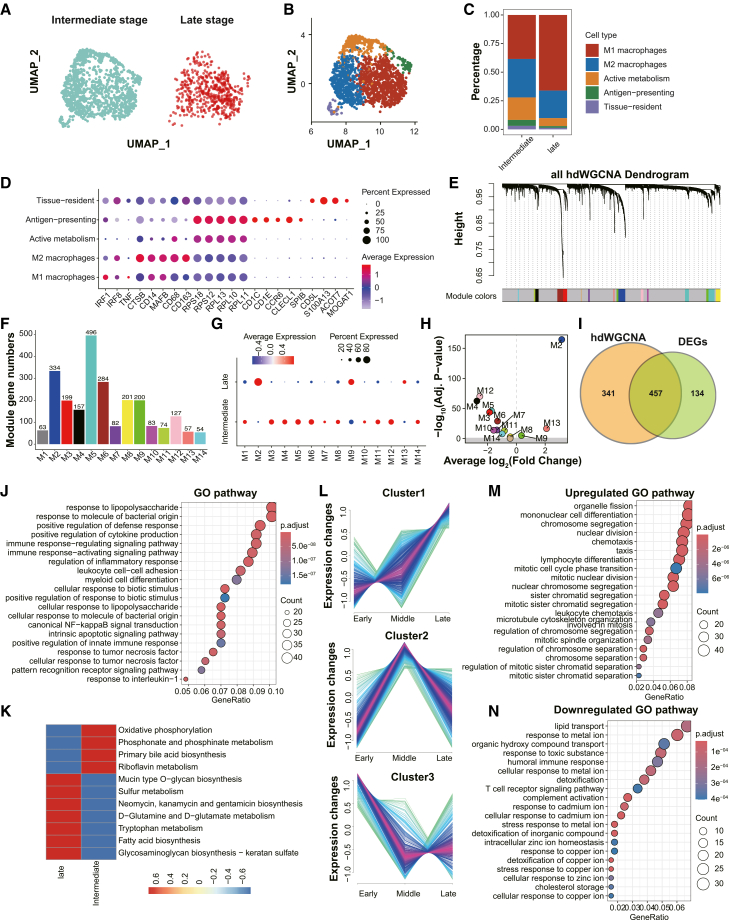
Transcriptional and metabolic reprogramming of macrophages during lung injury progression (A) UMAP visualization of macrophages isolated from intermediate-severity and late-severity BALF samples. (B) UMAP projection shows five macrophage subtypes annotated by marker gene expression: M1 macrophages, M2 macrophages, metabolically active macrophages, antigen-presenting macrophages, and tissue-resident macrophages. (C) Proportional distribution of macrophage subtypes between intermediate and late stages. Late-severity samples are enriched for M1 macrophages, while M2, antigen-presenting, metabolically active, and tissue-resident subtypes decline. (D) Dot plot displays average expression and percent expression of representative genes across macrophage subtypes. (E) Hierarchical clustering dendrogram generated by hdWGCNA of macrophages, identifying 14 distinct co-expression modules based on transcriptional similarities. (F) Bar plot shows the number of genes per module. (G) Dot plot shows average expression of each module across intermediate and late stages. M2, M9, and M13 are enriched in the late stage. (H) Volcano plot of differential expression of module eigengenes between stages. (I) Venn diagram showing overlap between late-severity DEGs and genes in upregulated hdWGCNA modules. (J) GO enrichment of overlapping genes from (I), revealing pathways such as LPS response, cytokine production, TNF signaling, and NF-κB activation. (K) scMetabolism-based analysis of enriched metabolic pathways in macrophages at intermediate and late stages. (L) Mfuzz clustering of a public macrophage dataset (GSM4475050) identifies three gene clusters with distinct temporal expression patterns across early, middle, and late stages. (M) GO enrichment of genes upregulated over time, indicating increased cell proliferation and lymphocyte differentiation. (N) GO enrichment of genes downregulated over time, reflecting decreased immune regulation, detoxification, and lipid transport.

To elucidate the transcriptional heterogeneity and functional dynamics of macrophages during lung injury severity escalation, we conducted subclustering analysis on macrophages derived from intermediate- and late-severity BALF samples. Through unsupervised UMAP embedding and marker-guided annotation, five macrophage subtypes were identified: M1 macrophages, M2 macrophages, metabolically active macrophages, antigen-presenting macrophages, and tissue-resident macrophages (Figures 5A–5D). Notably, M1 macrophages—characterized by the elevated expression of IRF1, TNF, and IRF8—were markedly expanded in late-severity samples. In contrast, M2 macrophages (e.g., CTSB and CD163), antigen-presenting macrophages (e.g., CD1C and CLECL1), metabolically active macrophages (e.g., RPS and RPL family), and tissue-resident macrophages (e.g., CD5L, S100A13, and ACOT7) were reduced in late-severity samples, suggesting a phenotypic shift toward proinflammatory polarization and a depletion of reparative subsets (Figures 5C and 5D).

Subsequently, to investigate transcriptional coordination across macrophage subsets, we applied high-dimensional weighted gene co-expression network analysis (hdWGCNA), which identified 14 distinct gene modules across the macrophage population (Figures 5E and 5F). Among them, modules M2, M9, and M13 were enriched in late-severity macrophages, indicating co-regulated inflammatory programs (Figures 5G and 5H). Importantly, these modules showed considerable overlap with upregulated DEGs from late-severity macrophages (Figure 5I), underscoring their relevance in capturing core inflammatory responses. GO enrichment analysis of the intersected gene sets further revealed activation of canonical proinflammatory pathways, including response to lipopolysaccharide, TNF, IL-1 signaling, NF-κB activation, leukocyte adhesion, and innate immune regulation (Figure 5J).

To assess macrophage metabolic states across severity strata, we employed the scMetabolism algorithm.^28^ Compared to their late-severity counterparts, intermediate-severity macrophages exhibited greater inferred activity in oxidative phosphorylation, phosphonate and phosphate metabolism, and bile acid biosynthesis (Figure 5K), indicative of higher transcriptome-inferred mitochondrial respiratory programs associated with immune surveillance and tissue maintenance. Conversely, late-severity macrophages showed increased enrichment in mucin-type O-glycan biosynthesis, sulfur metabolism, glutamate metabolism, and glycosaminoglycan biosynthesis—pathways associated with immunosuppressive remodeling, extracellular matrix interactions, and barrier regulation. These results suggest a shift in metabolic programming associated with increasing severity, from energy-demanding immune effector states toward context-specific remodeling phenotypes during late-severity lung injury.

Furthermore, to evaluate macrophage activation patterns across healthy and injury conditions, we incorporated a public scRNA-seq dataset of healthy alveolar macrophages (GSM4475050)^16^^,^^29^ and applied Mfuzz-based temporal clustering across healthy, intermediate-severity, and late-severity phases (Figure 5L).^30^^,^^31^ Clusters enriched in late-severity samples exhibited gene signatures involved in mitotic regulation and immune cell differentiation, such as chromosome segregation, nuclear division, and lymphocyte development (Figure 5M), indicating proliferative and immune-activating transitions. In contrast, genes downregulated along the condition-ordered trajectory were linked to lipid transport, metal ion response, complement activation, and humoral immunity (Figure 5N), reflecting a progressive loss of immunoregulatory and detoxification capacities.

Taken together, these findings reveal a severity-dependent reprogramming of macrophage populations during lung injury, transitioning from diverse homeostatic and immunoregulatory functions in the intermediate stage to a proinflammatory and metabolically reprogrammed phenotype in late-severity disease. This transcriptional and metabolic remodeling likely contributes to the impaired resolution of inflammation and the persistence of tissue injury in advanced lung injury.

### A granulocyte-centric TNF/IFN/RESISTIN signaling network dominates the late-severity immune microenvironment

**Figure 6:**
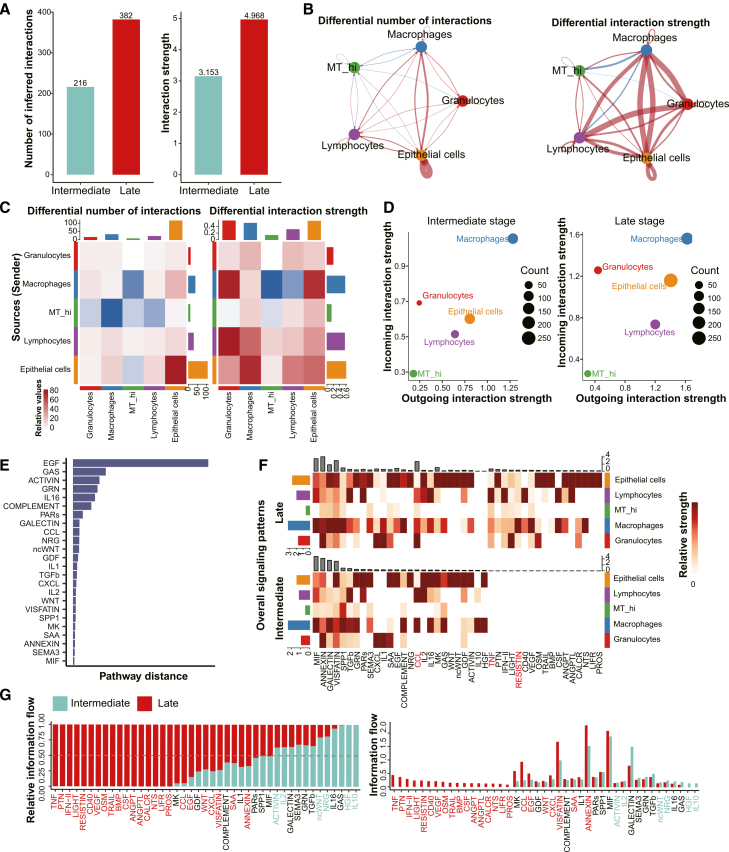
System-wide remodeling of intercellular communication networks during lung injury progression (A) Bar plots show the total number of inferred ligand-receptor interactions (left) and overall interaction strength (right) in intermediate-severity and late-severity BALF samples, as inferred by CellChat. The total cell communication increased from 216 to 382, and the intensity increased from 3.153 to 4.968. Intermediate-severity is represented in turquoise and late-severity in red. (B) Circular diagrams depict the number of receptor-ligand pairs and communication action quantities (left) as well as their intensities (right) between different cell populations in the intermediate-severity versus the late-severity. Here, red edges indicate interactions that increased with the late-severity group, while blue edges represent those that decreased. (C) Heatmap displays differential interaction intensities across cell types. The top bar summarizes incoming signal strength per cell type (sum of the columns), while the right bar represents outgoing signal strength (sum of the rows). In the color bar, red indicates increased interactions in the late-severity group compared to the intermediate-severity group, and blue indicates decreased interactions. (D) Dot plots illustrate changes in cell-to-cell communication. Comparisons in a 2D space of outgoing and incoming interaction intensities identify cell populations with significant changes in sending or receiving signals between late- and intermediate-severity. The horizontal axis represents the strength of efferent interactions (i.e., the cell’s ability to send signals), and the vertical axis represents the strength of incoming interactions (i.e., the cell’s ability to receive signals). Different cell types are represented by different colors, and their size represents cell abundance. The left and the right correspond to the intermediate-severity and late-severity groups, respectively. (E) Identification of significantly different signal networks based on the Euclidean distance in a shared bi-dimensional space. A larger distance implies a more significant functional difference between the communication networks in the two stages. Only the distance between overlapping signal pathways in both datasets is calculated. (F) Heatmaps compare the overall signaling patterns in immune cell populations between the intermediate-severity and late-severity BALF samples. Rows represent individual signaling pathways, and columns correspond to distinct immune cell types. Color intensity reflects the relative contribution score of the respective cell type to each signaling pathway. The relative signaling strength was derived from pattern recognition analysis, with higher scores indicating stronger contributions to specific signaling pathways. (G) Bar graphs show relative (left) and absolute (right) information flow of each signaling pathway across stages. Red bars highlight pathways upregulated in the late stage; turquoise bars indicate those dominant in the intermediate stage.

To explore severity-associated remodeling of cell-cell communication during the progression of lung injury, we conducted intercellular interaction analyses using CellChat across five principal immune and epithelial compartments.^32^^,^^33^ Notably, both the number of inferred intercellular interactions and their aggregate communication strength were elevated in late-severity samples relative to intermediate-severity samples, indicating intensified crosstalk among cell populations with increasing severity (Figure 6A). Visualization of communication networks pinpointed that this enhancement was primarily driven by augmented interactions among granulocytes, macrophages, and epithelial cells (Figure 6B), with granulocytes becoming increasingly central to both outgoing and incoming signaling activities in the late-severity stratum.

Further analysis using sender-receiver heatmaps revealed that granulocytes and macrophages showed increased inferred outgoing signaling toward epithelial and lymphoid compartments during late-severity injury (Figure 6C). These results suggest that innate immune cells not only may reinforce autocrine signaling but also may amplify paracrine signaling to adjacent cell types in a severity-associated manner. Complementary scatterplot analyses showed that granulocytes and epithelial cells evolved into dominant signaling hubs, while macrophages, though reduced in number, maintained substantial signaling output (Figure 6D).

To dissect the molecular drivers of these network changes, we performed ligand-receptor pathway analysis. EGF, GAS, ACTIVIN, and GRN emerged as prominent mediators of signaling reconfiguration (Figure 6E), consistent with their documented roles in epithelial remodeling, immune regulation, and inflammation.^34^ Late-severity samples exhibited expanded engagement of proinflammatory and proliferative signaling axes, with the pronounced activation of CCL, TNF, and RESISTIN pathways (Figure 6F). To ensure statistical robustness, we explicitly applied a permutation-based framework to assess communication probability, retaining only ligand-receptor pairs with permutation-based interaction strength (permutation *p* < 0.05) for all downstream analyses (Table S3).

Pathway-level comparisons revealed a marked upregulation of TNF, IFN, and LIGHT signaling during late-severity lung injury (Figure 6G, left). Among these, TNF, IFN, and RESISTIN pathways demonstrated prominent overall information flow, consistent with a central role in predicted network amplification and persistent inflammation (Figure 6G, right). To further validate these patterns at the molecular level, we visualized specific ligand-receptor pairs using bubble plots (Figure S4). While CCL, MIF, and SPP1 signaling maintained significant interactions across major compartments in both severity strata (Figures S4A–S4C), the relative increase of TNF, RESISTIN, and LIGHT signaling was driven by late-severity-specific ligand-receptor pairs (e.g., TNF-TNFRSF1A/B and RETN-CAP1) that were statistically significant in late-severity disease but notably absent or non-significant in the intermediate stage (Figures S4D–S4F). In contrast, HGF, IL10, and ncWNT signaling were more pronounced in intermediate-severity samples, potentially reflecting a transient reparative or immunoregulatory milieu during earlier disease stages.

Collectively, these analyses highlight a dramatic reorganization of inferred intercellular signaling networks associated with increasing severity. The emergence of granulocyte-centric signaling, particularly via TNF superfamily members and chemokine-driven pathways, suggests a putative self-perpetuating inflammatory circuit. Such a predicted loop may underlie the transition from acute injury to chronic tissue damage and fibrotic remodeling, providing mechanistic insight into the sustained immune activation observed in late-severity disease.

### *In vivo* validation confirms the causality of TNF/MAPK signaling and metabolic reprogramming in driving late-severity ALI

**Figure 7:**
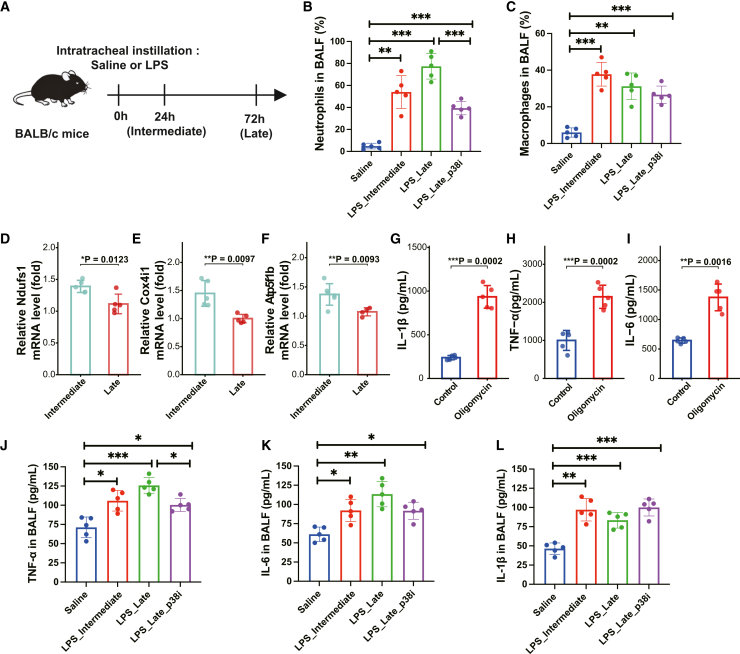
*In vivo* validation confirms that metabolic impairment and TNF/MAPK signaling drive late-severity ALI pathology (A) Schematic of the experimental design. BALB/c mice received intratracheal instillation of saline or LPS (4 mg/kg) and were analyzed at 24 h (Intermediate) and 72 h (Late) to mimic disease progression. For intervention, a subset of mice received a p38 MAPK inhibitor (p38i) treatment during the late phase. (B and C) Flow cytometric analysis of immune cell dynamics. Quantification of the percentage of (B) neutrophils (Ly6G^+^) and (C) macrophages (F4/80^+^) in murine BALF across groups. Consistent with human data, neutrophil proportions expand significantly in the late stage (reversed by p38i), while macrophage proportions significantly decline in the late stage compared to the intermediate stage. (D–F) Validation of metabolic reprogramming. Relative mRNA expression of representative oxidative phosphorylation (OXPHOS)-related genes, including (D) *Ndufs1*, (E) *Cox4i1*, and (F) *Atp5f1b*, in sorted murine alveolar macrophages. Data show a significant downregulation in the late stage compared to the intermediate stage, confirming OXPHOS impairment. (G–I) Causal validation of metabolic-driven inflammation. Primary alveolar macrophages were isolated from 24 h LPS-treated mice (Intermediate stage) and treated with the ATP synthase inhibitor Oligomycin (1 μM) or Vehicle (Control) for 6 h. The concentrations of (G) IL-1β, (H) TNF-α, and (I) IL-6 in the culture supernatants were quantified by ELISA. Inhibition of OXPHOS significantly increased cytokine secretion, mimicking the late-severity hyper-inflammatory phenotype. (J–L) Causal validation of the TNF/MAPK signaling axis. ELISA quantification of (J) TNF-α, (K) IL-6, and (L) IL-1β in murine BALF. The elevated cytokine storm in late-severity ALI is significantly dampened by p38 MAPK inhibition. Data are presented as mean ± SD (*n* = 5). Statistical significance was determined by Student’s *t* test (two groups) or one-way ANOVA with Tukey’s post-hoc test (multiple groups); ∗*p* < 0.05, ∗∗*p* < 0.01, and ∗∗∗*p* < 0.001.

To assess the biological plausibility and mechanistic causality of our single-cell inferences *in vivo*, we established a time-resolved intratracheal LPS-induced murine ALI model. BALB/c mice were analyzed at 24 h (intermediate) and 72 h (late) post-injury to mimic intermediate-like and late-like injury phases (Figure 7A).

First, we sought to determine whether the immune landscape remodeling identified in human ALI is conserved in mice. Flow cytometric analysis of murine BALF revealed a significant expansion of Ly6G^+^ neutrophils in the late stage compared to the intermediate stage (Figure 7B). In parallel, we observed a significant reduction in the proportion of F4/80^+^ macrophages in the late-stage relative to the intermediate stage (Figure 7C). These distinct cellular shifts accurately recapitulate the granulocyte expansion and macrophage depletion observed in our human cohort.

Concurrently, we validated the metabolic reprogramming signature identified in our scMetabolism analysis. RT-qPCR of sorted late-severity murine macrophages demonstrated a significant downregulation of key oxidative phosphorylation (OXPHOS)-related genes (e.g., *Ndufs1*, *Cox4i1*, and *Atp5f1b*) compared to the intermediate stage (Figures 7D–7F). This confirms that the impairment of OXPHOS is a conserved feature of late-severity macrophages.

Next, to determine whether this metabolic shift is causally linked to the sustained inflammation, we performed an *in vitro* interventional experiment (mimicry experiment). We isolated primary alveolar macrophages from the intermediate stage—which exhibit relatively preserved metabolic function—and treated them with Oligomycin (an ATP synthase inhibitor) to experimentally mimic the OXPHOS defect observed in the late stage. Strikingly, pharmacological inhibition of OXPHOS significantly upregulated the secretion of pro-inflammatory cytokines, including IL-1β, TNF-α, and IL-6 (Figures 7G–7I). These results provide direct evidence that metabolic reprogramming—specifically the loss of mitochondrial respiration—acts as a driver of the hyper-inflammatory phenotype.

Finally, to demonstrate the causal role of the granulocyte-centric TNF/MAPK signaling network predicted by our CellChat analysis, we blocked the p38 MAPK pathway, a crucial downstream effector of TNF signaling, in the murine model. Indeed, treating mice with a p38 MAPK inhibitor (p38i) during the injury phase significantly reduced the percentage of neutrophils in BALF (Figure 7B) and markedly attenuated the cytokine storm, as evidenced by reduced levels of TNF-α, IL-6, and IL-1β in the bronchoalveolar space (Figures 7J–7L).

Collectively, these *in vivo* and *in vitro* data provide strong experimental confirmation that macrophage metabolic reprogramming (impaired OXPHOS) and the granulocyte-centric TNF/MAPK signaling axis are functionally essential drivers of persistent inflammation in late-severity ALI.

## Discussion

In this study, we provide a comprehensive single-cell transcriptomic atlas of the immune microenvironment across intermediate and late stages of ALI. Unlike most classical and contemporary studies that have characterized neutrophils and macrophages either in bulk, at a single time point, or in isolation,^1^^,^^15^ our work delineates the dynamic co-evolution of these lineages within the same analytical framework. (Figure 1-1). Through scRNA-seq combined with detailed computational analyses, including pseudotime trajectories, co-expression network analysis (hdWGCNA), and metabolic modeling (scMetabolism), we identified stage-specific activation programs in granulocytes and macrophages that mechanistically distinguish intermediate-severity containment from late-severity immunopathology.

A pivotal finding from our analysis is the persistent expansion of granulocytes and concomitant depletion of macrophages in late-severity ALI (Figure 1-2). While prior single-cell atlases have described immune heterogeneity in ARDS or COVID-19,^16^^,^^17^^,^^18^ our specific focus on the intermediate-to-late transition reveals a granular, stage-dependent failure of resolution. We demonstrate that late-severity ALI is not merely a prolongation of early inflammation but represents a distinct state characterized by a granulocyte-centric inflammatory network.^35^^,^^36^ Granulocyte trajectory analysis demonstrates pseudotemporal progression from chemotactic activation (cluster 1), through highly inflammatory (cluster 2, enriched in TNF, MAPK, and IL-1 pathways), stress-adapted (cluster 3), and immunoregulatory states (cluster 4). Critically, we show that these late-severity granulocytes serve as major signaling hubs, broadcasting TNF-, IFN-, and RESISTIN-linked signals to macrophages and epithelial cells, accompanied by metabolic shifts from lipid and glycan biosynthesis to amino acid and redox metabolism, reflecting sustained inflammatory activation (Figure 1-3).

Macrophages undergo distinct phenotypic reprogramming, characterized by the depletion of antigen-presenting, metabolically active, and reparative (M2-like) subsets in late-severity ALI, with the concurrent enrichment of proinflammatory M1-like subsets (Figure 1-4). Co-expression network and metabolic analyses revealed that intermediate-severity macrophages predominantly exhibit oxidative phosphorylation, phosphonate, and bile-acid metabolism, supporting immune surveillance and tissue homeostasis. In contrast, late-severity macrophages engage mucin-type O-glycan biosynthesis, sulfur, and glutamate metabolism, consistent with proinflammatory and fibrotic activation. This transition underscores a disease-severity-specific functional reprogramming of macrophage metabolism and phenotype.

Furthermore, significant remodeling of intercellular communication networks was observed during ALI progression (Figure 1-5). CellChat analysis revealed enhanced ligand-receptor interactions primarily driven by granulocytes and macrophages, with notable amplification of TNF, RESISTIN, and IL-1 pathways, establishing a sustained feedforward inflammatory network. Concurrently, suppression of reparative signaling pathways (GAS, ncWNT) alongside selective enhancement of pro-inflammatory cues underscores the transition from resolution-oriented signaling toward chronic inflammation.

Importantly, we validated these single-cell inferences using primary human cells and a time-resolved murine ALI model. RT-qPCR analysis confirmed the stage-specific reprogramming of key inflammatory and lysosomal genes in both species. Notably, these *in vivo* dynamics mirror the persistent pathology described in “two-hit” severe pneumonia models,^37^ reinforcing the biological plausibility of the non-resolving, granulocyte-centric circuit identified in our human cohort.

Collectively, our findings elucidate stage-dependent immune reprogramming mechanisms underlying persistent inflammation and tissue injury in ALI, providing a detailed mechanistic understanding and suggesting therapeutic targets, including TNF blockade, RESISTIN neutralization, and metabolic modulators, to restore immune homeostasis and mitigate disease progression (Figure 1-6). These mechanistic insights suggest that immunomodulation must be strictly stage-adapted. While we identify TNF blockade and RESISTIN neutralization as potential strategies to disrupt the self-perpetuating myeloid loops, we caution that the systemic inhibition of these central mediators carries risks of immunosuppression, particularly in patients with septic.^38^ Therefore, future interventions might prioritize lung-targeted delivery or patient stratification based on hyperinflammatory endotypes.

Beyond targeting specific cytokines, our analysis suggests that the distinct inflammatory modules identified here—specifically the amplification of TNF/IL-1 signaling and macrophage metabolic reprogramming—are likely sustained by upstream TLR/NF-κB activation and redox dysregulation. Interestingly, this pathogenic axis aligns with emerging preclinical evidence on Traditional Chinese Medicine (TCM). Recent studies indicate that TCM-derived polysaccharides (e.g., from Tetrastigma hemsleyanum) and formulae (e.g., Fuzhengjiedu and Guben Qingfei) attenuate LPS-induced lung injury by downregulating the TLR4/COX-2/NF-κB axis^39^^,^^40^ and enhancing Nrf2-mediated antioxidant defenses.^41^^,^^42^ These effects mirror the reversal of the specific pathogenic modules—granulocyte NF-κB activation and macrophage redox imbalance—that we mapped in human late-severity ALI, suggesting that such multi-component therapies may offer a valid approach to reshape these granulocyte-centric inflammatory networks.

Finally, our work provides clinically meaningful insights by revealing how granulocyte-driven inflammatory amplification and macrophage functional reprogramming characterize distinct stages of ALI progression. These findings underscore the importance of aligning therapeutic strategies with disease stage: For example, inhibiting granulocyte-mediated cytokine storms in early ALI or reactivating reparative macrophage programs in late-severity disease. The specific transcriptional signatures identified here could serve as biomarkers to distinguish patients on a regressive trajectory from those progressing toward fibrosis. Temporal profiling of immune dynamics thus offers a path toward rational, stage-specific interventions aimed at improving efficacy and minimizing harm.

In conclusion, our single-cell analysis charts the dynamic transitions of key innate immune cells in ALI and reveals stage-specific pathological mechanisms that drive disease progression. By highlighting the functional divergence and crosstalk of granulocytes and macrophages, our study offers a mechanistic foundation for precision immunotherapies tailored to ALI’s temporal landscape. These insights provide a theoretical framework for guiding targeted drug development, improving diagnostic accuracy, and informing clinical decisions, ultimately contributing to better patient outcomes in acute and progressive lung injury.

### Limitations of the study

While our study provides deep mechanistic insights into the stage-specific roles of granulocytes and macrophages in ALI, several limitations should be acknowledged from a clinical perspective. First, although our cohort captured intermediate and late stages of the disease, the cross-sectional sampling design limits the ability to monitor dynamic immune transitions within individual patients. Early-severity patients were not included due to the ethical and logistical constraints of performing bronchoscopy in potentially unstable, early-phase patients. While we integrated external healthy macrophage data^16^ to infer baseline deviations, the lack of longitudinal sampling means the described trajectories reflect pseudotemporal inferences. Second, our cohort specifically enrolled patients with bacterial pneumonia-associated ALI complicated by sepsis to reduce etiologic heterogeneity. Therefore, the generalizability of the described granulocyte-centric network to other ALI etiologies (e.g., viral pneumonia and trauma) remains to be verified in broader patient cohorts. Additionally, while our cohort reflects standard-of-care ICU management, we acknowledge that we cannot fully disentangle the potential influence of concurrent medications (e.g., antibiotics and corticosteroids) from disease-intrinsic immune remodeling. Moreover, given the pooled library design, cell-level statistics should be interpreted as a hypothesis-generating framework, which we strengthened by validating key metabolic and signaling findings through independent *in vivo* and *in vitro* assays. Finally, although our dataset encompasses major immune populations, rare or spatially restricted cell types may be underrepresented. Integrating spatial transcriptomics and protein-level validation techniques (e.g., CITE-seq) may help to further resolve microanatomical contexts and functional states.^43^

## Resource availability

### Lead contact

Further information and requests for resources and reagents should be directed to and will be fulfilled by the lead contact, Zhongshan He (zhongshan_he@163.com).

### Materials availability

This study did not generate new unique reagents.

### Data and code availability

• Data: The scRNA-seq datasets generated in this study have been deposited in the NCBI Gene Expression Omnibus (GEO) under accession GSE315604. Raw sequencing reads are available via the Sequence Read Archive (SRA) linked to this GEO record (BioProject PRJNA1398318). Processed gene-cell count matrices and accompanying sample metadata are provided through GEO.
• Code: This paper does not report original code.
• Additional information: Any remaining materials are available from the corresponding author upon reasonable request.

## Acknowledgments

This work is supported by the Youth Fund of the 10.13039/501100001809National Natural Science Foundation of China (no. 82400537, Z.H.), the Sichuan Science and Technology Program (no. 2024NSFSC0714, Z.H., no. 2025ZNSFSC0712, Y.F., and no. 2026NSFSC0562, C.C.), the 10.13039/501100020207Health Commission of Sichuan Province Medical Science and Technology Program (grant no. 25LCYJ42, Y.F.), the Postdoctoral Fellowship Program of CPSF under Grant (no. GZC20241133, Z.H.), the Postdoctoral Research and Development Program of 10.13039/501100004912Sichuan University (no. 2024SCU12014, Z.H.), and 10.13039/501100013365West China Hospital (no. 2024HXBH058, Z.H.).

## Author contributions

W.Z., C.W., and C.C. performed most of the experiments, conducted data analysis, and drafted the manuscript. Z.H., Y.F., and C.N. conceived and supervised the project. Z.H., and Y.Z. coordinated clinical sample collection and interpretation. Z.H. and Y.Z provided critical revision of the manuscript. All authors discussed the results and approved the final version of the manuscript.

## Declaration of interests

The authors declare no competing interests.

## STAR★Methods

### Key resources table

**Table:** 

REAGENT or RESOURCE	SOURCE	IDENTIFIER
**Antibodies**		

TruStain FcX™ (anti-mouse CD16/32)	BioLegend	Cat# 101320; RRID:AB_1574975
Anti-mouse CD45 (Clone 30-F11)	BioLegend	Cat# 103107; RRID:AB_312972
Anti-mouse Ly6G (Clone 1A8)	BioLegend	Cat# 127605; RRID:AB_1236488
Anti-mouse F4/80 (Clone BM8)	BioLegend	Cat# 123107; RRID:AB_893500
Anti-human CD45 (Clone HI30)	BioLegend	Cat# 304054; RRID:AB_2564154
Anti-human CD66b (Clone G10F5)	BioLegend	Cat# 305103; RRID:AB_314495
Anti-human CD68 (Clone Y1/82A)	BioLegend	Cat# 333805; RRID:AB_1089055

**Biological samples**		

Human BALF samples (ICU patients with ALI/ARDS)	West China Hospital, Sichuan University	N/A
Primary human granulocytes and macrophages	West China Hospital, Sichuan University	N/A

**Chemicals, peptides, and recombinant proteins**		

Collagenase IV	Sigma-Aldrich	N/A
DNase I	Roche	N/A
Dispase II	Sigma-Aldrich	N/A
ACK lysing buffer	Thermo Fisher Scientific	Cat# A1049201
Lipopolysaccharide (LPS)	Sigma-Aldrich	Cat# L4391
SB203580 (p38 MAPK inhibitor)	Selleck	Cat# S1076
Oligomycin A	Sigma-Aldrich	Cat# 75351
DMSO	Sigma-Aldrich	Cat# D2650
TRIzol reagent	Invitrogen	Cat# 15596026
DAPI	Thermo Fisher Scientific	Cat# D1306
Fetal bovine serum (FBS)	Gibco	N/A

**Critical commercial assays**		

Chromium Single Cell 3′ Reagent Kits v3.1	10x Genomics	N/A
PrimeScript RT Reagent Kit	Takara	N/A
SYBR Green Master Mix	Takara	N/A
Mouse IL-6 ELISA kit	Solarbio	Cat# SEKM-0007
Mouse TNF-α ELISA Kit	Solarbio	Cat# SEKM-0034
Mouse IL-1β ELISA Kit	Solarbio	Cat# SEKM-0002

**Deposited data**		

The raw single-cell RNA sequencing data	This study	Accession number: GEO: GSE315604; BioProject: PRJNA1398318

**Experimental models: Organisms/strains**		

BALB/c mice	Fukang Hua Biotechnology	N/A

**Oligonucleotides**		

Primers for qRT-PCR (human and mouse)	This study	Table S4

**Software and algorithms**		

Cell Ranger (v6.0.1)	10x Genomics	https://support.10xgenomics.com/
R (v4.3.0)	R Core Team	https://www.r-project.org
ClusterProfiler (v4.10.0)	Bioconductor	https://bioconductor.org/packages/release/bioc/html/clusterProfiler.html
KOBAS (v3.0)	Peking University (CBI)	http://kobas.cbi.pku.edu.cn
GraphPad Prism (v8.0)	GraphPad	https://www.graphpad.com
ggplot2 (v3.4.1)	CRAN	https://cran.r-project.org/web/packages/ggplot2/
DoubletFinder (v2.0.4)	GitHub	https://github.com/chris-mcginnis-ucsf/DoubletFinder
Monocle2 (v2.30.0)	Trapnell Lab	http://cole-trapnell-lab.github.io/monocle-release/
hdWGCNA	GitHub	https://github.com/smorabit/hdWGCNA
scMetabolism (v0.2.1)	GitHub	https://github.com/wu-lab/scMetabolism
Mfuzz (v2.62.0)	Bioconductor	https://bioconductor.org/packages/Mfuzz/
CellChat (v1.6.1)	GitHub	https://github.com/sqjin/CellChat
FlowJo	Tree Star	https://www.flowjo.com/

**Other**		

BD FACSAria™ Fusion sorter	BD Biosciences	N/A
BD FACSymphony flow cytometer	BD Biosciences	N/A
Illumina NovaSeq 6000 platform	Illumina	N/A

### Experimental model and study participant details

#### Study population

We enrolled seven adult ICU patients at West China Hospital, Sichuan University, with pneumonia-associated acute lung injury within the clinical spectrum of ARDS (Berlin framework), requiring ventilatory support. All subjects fulfilled the Berlin definition of ARDS at the time of bronchoscopy (acute onset, bilateral opacities on chest imaging, and respiratory failure not fully explained by cardiac failure or fluid overload). At BALF sampling, all cases corresponded to moderate–severe hypoxemia within the Berlin severity framework (PaO_2_/FiO_2_ ≤ 200 mmHg under PEEP/CPAP ≥ 5 cmH_2_O). Where available, clinical assessment and echocardiography and/or hemodynamic measurements supported the absence of hydrostatic edema; pulmonary artery wedge pressure values (if measured) are reported in Table S1 but were not used as a diagnostic threshold. Patients were stratified into an intermediate-severity group (*n* = 4) and a late-severity group (*n* = 3) according to APACHE II scores at the time of BALF sampling, reflecting systemic disease severity rather than calendar time from symptom onset. BALF was collected at a single clinically indicated time point per patient, and “days from onset” was not used as a formal stratification variable. Exclusion criteria included known severe chronic immunodeficiency, malignancy, or long-term use of potent immunosuppressive therapy to minimize baseline immune confounders. We did not exclude all chronic comorbidities typical of ICU populations; instead, comorbidities, acute complications, and key medications administered around BALF collection were recorded and are summarized in Table S1. Both sexes were included; however, due to the limited cohort size, sex-stratified analyses were not performed, and potential sex-associated effects are therefore not determined in this study.

#### Ethical approval statement

All human sample collection and analyses were conducted in accordance with the ethical standards of the Research Ethics Committee of West China Hospital, Sichuan University. Ethical approval was granted under Approval Number 2023-193. Written informed consent was obtained from all participants prior to sample collection and data analysis. Additionally, All animal experiments were carried out with the guidelines of the Institutional Animal Care and Use Committee (IACUC) of Sichuan University, with prior approval (Ethics Approval Number: 20221110001).

### Method details

#### Sample collection and preparation

BALF samples were obtained during clinically indicated bronchoscopy following standardized ICU protocols approved by the institutional ethics committee. To preserve cell viability and RNA integrity, BALF was transported on ice and processed immediately after collection. Samples were filtered to remove mucus and debris, and cells were collected by centrifugation. Where required, red blood cells were removed using ACK lysis buffer, followed by washing in PBS (or PBS supplemented with BSA, as used downstream). Cell viability was assessed prior to downstream applications. Aliquots of cell-free BALF supernatant were generated by additional centrifugation and stored at −80°C for cytokine measurements. For scRNA-seq, leukocytes were enriched and prepared as single-cell suspensions for downstream sorting and encapsulation (detailed below). For sequencing library construction, BALF-derived cells were pooled within each severity group to increase cell yield and reduce technical variability across individual samples, resulting in one pooled “Intermediate” library and one pooled “Late” library. While pooling precludes subject-level attribution of single-cell profiles, it allows for robust group-level characterization of the immune landscape.

#### Isolation and preparation of single-cell suspensions

BALF samples underwent gentle mechanical dissociation followed by enzymatic digestion in RPMI 1640 medium containing 1 mg/mL collagenase IV (Sigma-Aldrich), 50 U/mL DNase I (Roche), and 1 U/mL dispase II (Sigma-Aldrich) for 30 min at 37°C with gentle agitation. Digested samples were filtered through a 70 μm nylon mesh to remove debris, and cells were pelleted by centrifugation at 300 × g for 5 min at 4°C. Red blood cells were lysed using ACK lysing buffer (Thermo Fisher Scientific) for 3 min, followed by two washes in PBS containing 0.04% BSA. Cells were resuspended in PBS with 0.04% BSA, stained with DAPI (Thermo Fisher Scientific) to assess viability, and counted to determine cell concentration prior to downstream applications. To minimize aggregates and reduce the risk of microfluidic clogging, the suspension was kept on ice and, when necessary, passed through an additional cell strainer immediately before loading.

#### Single-cell RNA-sequencing (scRNA-seq) library preparation

To ensure sufficient cell yield for library construction and mitigate inter-individual technical variability, single-cell suspensions were pooled within each group prior to encapsulation. Specifically, cells from the four intermediate-severity patients were pooled to generate one “Intermediate” library, and cells from the three late-severity patients were pooled to generate one “Late” library. Samples were processed using the 10× Genomics Chromium Single Cell 3’ Reagent Kits v3.1 according to the manufacturer’s instructions. Approximately 8,000-10,000 cells per sample were loaded onto each Chromium chip channel to generate single-cell gel bead-in-emulsions (GEMs). Reverse transcription, cDNA amplification, and library construction were performed following the standard protocol. Libraries were sequenced using the Illumina NovaSeq 6000 platform with a read configuration of 28 bp for read 1 (cell barcode and UMI), 8 bp for the i7 index, and 91 bp for read 2 (transcript sequence).

#### Murine ALI model and treatment

To validate stage-dependent immune remodeling *in vivo*, a time-resolved LPS-induced ALI model was established in BALB/c mice. Male BALB/c mice (6–8 weeks old; 20–25 g) were purchased from Beijing Huafukang Biotechnology Co., Ltd. and acclimated for 1 week under specific pathogen–free conditions at Sichuan University with *ad libitum* access to food and water and a 12 h light/dark cycle. Mice were randomly assigned to experimental groups. LPS (Escherichia coli O111:B4; Sigma-Aldrich) was dissolved in sterile PBS at 4 mg/mL, and administered intratracheally at a dose of 4 mg/kg. Briefly, mice were anesthetized with isoflurane (Shenzhen RWD Life Science Co., Ltd.), the trachea was visualized using a small animal laryngoscope (HY-SHJ01), and LPS was delivered using a Liquid MicroSprayer (HY-LWH02; Beijing YSKD Biotechnology Co., Ltd.). Control mice received an equal volume of sterile PBS. Mice were euthanized at 24 h (intermediate phase) or 72 h (late phase) post-challenge for downstream BALF collection and tissue processing.

For pathway perturbation, a subset of LPS-challenged mice received the p38 MAPK inhibitor SB203580 (Selleck) at 10 mg/kg by intraperitoneal injection, prepared freshly in an appropriate vehicle according to the manufacturer’s recommendations. To target the late-phase inflammatory surge assessed at 72 h, SB203580 was administered during the injury course prior to the 72 h endpoint (e.g., at 24 h and 48 h post-LPS), while vehicle-treated mice received matched injections on the same schedule. All animal experiments were approved by the Animal Ethics Committee of West China Hospital.

#### Murine sample collection and processing

Bronchoalveolar lavage fluid (BALF) was collected from mice at the indicated time points by cannulating the trachea and lavaging the lungs three times with 1 mL of ice-cold phosphate-buffered saline (PBS). The recovered BALF was kept on ice and centrifuged at 300 × g for 5 min at 4°C to separate cells and supernatant. The cell pellet was used for downstream flow cytometry and cell sorting (see below). The supernatant (cell-free BALF) was transferred to new tubes, aliquoted, and stored at −80°C until cytokine measurements by ELISA. Repeated freeze–thaw cycles were avoided. For serum collection, whole blood was obtained from mice at the time of sacrifice, allowed to clot, and centrifuged to separate serum. Serum was aliquoted and stored at −80°C until ELISA analysis. All sample processing steps were performed on ice whenever possible to preserve protein integrity.

#### Flow cytometry and cell sorting

BALF cell pellets were resuspended in FACS buffer (PBS containing 2% fetal bovine serum). To minimize non-specific antibody binding, cells were incubated with Fc receptor blocking antibody TruStain FcX™ (BioLegend, Cat# 101320, 1 μg/mL) for 15 min on ice. Cells were then stained with fluorophore-conjugated antibodies for 30 min at 4°C in the dark, including anti-mouse CD45 (BioLegend, Clone 30-F11, 0.2 μg/mL) to identify leukocytes, anti-mouse Ly6G (BioLegend, Clone 1A8, 0.25 μg/mL) for neutrophils, and anti-mouse F4/80 (BioLegend, Clone BM8, 0.5 μg/mL) for macrophages. After staining, cells were washed twice with FACS buffer to remove unbound antibodies and resuspended in PBS containing DAPI (Thermo Fisher Scientific) to exclude dead cells. Flow cytometry data were acquired on a BD FACSymphony flow cytometer (BD Biosciences, San Jose, CA, USA) and analyzed using FlowJo software (Tree Star). The general gating strategy was as follows: debris exclusion by FSC/SSC, singlet gating (FSC-A vs FSC-H), live-cell gating (DAPI^-^), followed by identification of leukocytes (CD45^+^) and downstream annotation of neutrophils (CD45^+^Ly6G^+^) and macrophages (CD45^+^F4/80^+^). For downstream gene expression analysis, live CD45^+^Ly6G^+^ neutrophils and live CD45^+^F4/80^+^ macrophages were sorted using a BD FACSAria™ Fusion cell sorter directly into chilled collection tubes. Post-sort purity was confirmed and routinely exceeded 95%.

#### Enzyme-linked immunosorbent assay (ELISA)

The concentrations of inflammatory cytokines (TNF-α, IL-6, and IL-1β) in cell-free BALF, serum, and macrophage culture supernatants were quantified using commercial ELISA kits according to the manufacturer’s instructions. On the day of assay, all reagents and samples were equilibrated to room temperature. Standards were prepared by serial dilution to generate a standard curve on each plate, and samples were measured in duplicate. Where necessary, samples were diluted using the kit-provided sample diluent to ensure values fell within the linear range of the standard curve. Following sequential incubation with kit-provided capture/detection reagents and wash steps to minimize background, color was developed using TMB substrate and the reaction was terminated with stop solution. Absorbance was measured at 450 nm using a microplate reader (with background correction using a reference wavelength when applicable). Cytokine concentrations were calculated from the standard curve using four-parameter logistic (4PL) regression. Duplicate wells with excessive variability were re-assayed, and values below the lower limit of detection were handled according to the kit instructions. Results were reported as concentration per volume for BALF and serum, or per volume of culture supernatant for *in vitro* experiments.

#### Isolation of primary human cells and quantitative real-time PCR (qRT-PCR)

To validate the transcriptional signatures identified by scRNA-seq, primary human granulocytes and macrophages were isolated from fresh BALF samples obtained from patients with intermediate- and late-severity ALI. Briefly, cells were stained with fluorophore-conjugated antibodies and sorted via fluorescence-activated cell sorting (FACS) to purify neutrophils (CD45^+^CD66b^+^) and macrophages (CD45^+^CD68^+^). Total RNA was extracted from the sorted cells using TRIzol reagent (Invitrogen) according to the manufacturer’s instructions. Subsequently, RNA was reverse-transcribed into cDNA using the PrimeScript RT Reagent Kit (Takara). Quantitative real-time PCR was performed using SYBR Green Master Mix (Takara) on a CFX96 Touch Real-Time PCR Detection System (Bio-Rad). Gene-specific primers were used for amplification, and their sequences are listed in Table S4. Relative gene expression levels were calculated using the 2^−ΔΔCt^ method, normalized to GAPDH.

#### Pharmacological inhibition of oxidative phosphorylation in primary alveolar macrophages

To mechanistically validate the link between mitochondrial oxidative phosphorylation (OXPHOS) impairment and inflammatory phenotypes, primary alveolar macrophages were isolated from BALB/c mice at 24 h post-LPS instillation (intermediate stage). BALF cells were prepared as described above and live CD45^+^F4/80^+^ macrophages were purified by fluorescence-activated cell sorting (FACS). Sorted macrophages were seeded in 24-well plates at a density of 2 × 10^5^ cells per well in RPMI 1640 supplemented with 10% fetal bovine serum (Gibco). After 2 h of adherence at 37°C in a humidified 5% CO_2_ incubator, the medium was replaced to remove non-adherent cells. To mimic the late-severity OXPHOS defect, adherent macrophages were treated with Oligomycin A (Sigma-Aldrich, Cat# 75351; dissolved in DMSO) at a final concentration of 1 μM for 6 h. Control cells received an equal volume of vehicle (DMSO; Sigma-Aldrich, Cat# D2650). After treatment, culture supernatants were collected and centrifuged briefly to remove residual cells and debris. The clarified supernatants were aliquoted and stored at −80°C until cytokine measurement. The concentrations of IL-1β, TNF-α, and IL-6 in culture supernatants were quantified by ELISA (Wuhan jiyinmei biotech Co., Ltd.) as described in the ELISA section. All samples were measured in duplicate.

#### Preprocessing and quality control of scRNA-seq data

Raw sequencing reads were demultiplexed and aligned to the GRCh38 human reference genome using Cell Ranger (v6.0.1, 10× Genomics),^44^ and gene-cell expression matrices were generated using the count function. Initial Cell Ranger processing estimated 5,912 and 5,063 cells for the intermediate and late stages, respectively (Figure S1A). Downstream processing was performed with the Seurat package (v4.4.0) in R,^45^^,^^46^^,^^47^^,^^48^^,^^49^ where gene expression matrices were imported from output directories via the readMM function alongside barcode and feature annotations. Column and row names were assigned to cell barcodes and gene names, respectively. Each sample was initialized as a Seurat object using CreateSeuratObject, then filtered to retain cells expressing >200 genes, containing 500-50,000 total RNA counts, and exhibiting mitochondrial gene expression below 10% of total counts.

Following quality control, gene expression data underwent log-normalization (NormalizeData), and highly variable genes were identified with FindVariableFeatures before scaling via ScaleData. Putative doublets were detected and removed using the DoubletFinder package (v2.0.4).^50^ The optimal pK parameter was determined via paramSweep, summarizeSweep, and find.pK functions based on the bimodality coefficient (BCmvn). The expected number of doublets was estimated assuming a 7.5% doublet formation rate, adjusted by homotypic proportions inferred from clustering. Doublets were predicted using the doubletFinder function with 20 principal components and classified based on the optimized parameters (e.g., pN = 0.25, pK = 0.21).^51^ A total of 202 predicted doublets were removed. After rigorous quality control and doublet removal, a total of 10,504 high-quality singlet cells (5,551 intermediate-severity; 4,953 late-severity) were retained for downstream analyses.

Batch effects were corrected using Seurat’s reciprocal PCA-based integration workflow, where individual Seurat objects were merged via FindIntegrationAnchors across the top 30 principal components and integrated with IntegrateData to produce a unified, batch-corrected expression matrix. The integrated object was rescaled, subjected to PCA (RunPCA, 50 components), and the first 8 principal components were selected based on elbow plot inspection for clustering and visualization. Graph-based clustering was executed using FindNeighbors and FindClusters (resolution = 1.2), and cellular landscapes were visualized via UMAP dimensionality reduction (RunUMAP). All metadata, including sample identity and cluster assignments, were preserved in the integrated object for subsequent analysis.

#### Clustering and cell type annotation

Cell type identities were determined by canonical marker gene expression patterns within each cluster. Macrophages were characterized by C1QA, C1QB, C1QC, CD68, MRC1, TREM2, CSF1R, and CD163 markers. Granulocytes showed increased expression of S100A8, FCGR3B, ITGAM, MNDA, CD177, MME, C5AR1, and ITGA4, while lymphocytes were identified through CD3D, CD3E, CD8A, TRAC, CCL5, CD28, and KLRB1 expression. MT_hi cells displayed elevated expression of mitochondrial-encoded genes such as MT-ATP8, MT-ND6, MT-ND4L, MT-CO1, and MT-CYB. Epithelial cells were distinguished by CDH1, EPCAM, MUC1, KRT17, KRT8, KRT18, KRT19, CLDN3, and CLDN4 expression. These annotated clusters formed the basis for subsequent analyses.

#### Differential gene expression (DGE) analysis

Differential gene expression (DGE) analysis was performed at the single-cell level using the FindMarkers function in the Seurat package (v4.4.0), which applies the Wilcoxon rank-sum test. Given that scRNA-seq data are zero-inflated and do not follow a normal distribution, the non-parametric Wilcoxon test was selected over traditional parametric methods. Cells were grouped into intermediate-severity and late-severity. Differentially expressed genes were defined as those expressed in at least 25% of cells in either group (min.pct = 0.25) and exhibiting a minimum log2 fold change of 0.25 (logfc.threshold = 0.25). For cluster-specific analyses, comparisons were performed within each granulocytes subtype and macrophage subtype. P-values were adjusted for multiple testing using the Benjamini-Hochberg method to control the false discovery rate (FDR), and genes with adjusted p-values < 0.05 were considered significant. Given that cells within a pooled library are not independent biological replicates, p-values from cell-level DE were interpreted cautiously and primarily used to rank candidate genes and pathways for downstream validation.

#### Gene signature scoring

To assess immunological and functional states in single cells, we calculated module scores for predefined gene sets using Seurat’s AddModuleScore function (v4.4.0). These gene sets represented five key biological processes: immune inhibitory signaling, fibrotic remodeling, inflammatory response, chemokine activity, and antiviral defense, sourced from published literature,^52^ KEGG pathways, or the MSigDB database via the msigdbr package. Each score was derived by averaging expression values after subtracting background signals from control genes with matched expression levels. Violin plots and stratified boxplots illustrated score distributions across cell types and disease stages (intermediate vs. late). Statistical comparisons of module scores between cell populations were performed using the Wilcoxon rank-sum test, a non-parametric method appropriate for single-cell distributions.

The Immune Inhibitory Score integrated exhaustion markers (PDCD1, CTLA4, LAG3, TIGIT) and myeloid checkpoint genes (CD274).^53^ Fibrotic remodeling capacity was quantified through the Fibrosis Score, which combined extracellular matrix genes (COL1A1, FN1), profibrotic cytokines (TGFB1, IL13), and remodeling enzymes (MMP2, TIMP1). Innate immune activation was captured by the Inflammatory Response Score, incorporating phagocytosis-related (CD68, SIRPA), chemotactic (CXCL1), and metabolic markers (ARG1). Chemokine Activity Scores reflected migratory potential via ligand-receptor pairs (CCL2-CCR2, CXCL10-CXCR3), while the Antiviral Response Score quantified interferon-stimulated genes (IFIT1, OAS1, ISG15), pattern recognition receptors (TLR3, TLR7), and cytosolic sensors (RIG-I, MDA5). The specific gene sets used for scoring are provided in the Table S2. Comparative analyses of these scores across cell populations and time points delineated dynamic immune alterations in the bronchoalveolar niche during acute lung injury progression.

#### Lineage-specific subclustering and functional characterization of granulocytes and macrophages

To dissect intralineage heterogeneity and functional specialization, granulocytes and macrophages were independently extracted from the integrated Seurat object for secondary analysis. Cells were reprocessed through normalization (LogNormalize), variable feature selection (vst method, top 2,000 features), scaling, and dimensionality reduction using PCA. Clustering was performed with a resolution of 0.8 based on the top 15 principal components, followed by UMAP visualization to depict cellular distributions and subcluster relationships. Marker genes for each granulocyte or macrophage subcluster were identified using the FindAllMarkers function, and representative marker heatmaps were generated using DoHeatmap.

#### Pathway enrichment analysis

To elucidate the biological functions associated with differentially expressed genes (DEGs) and pseudotime-associated gene modules, Gene Ontology (GO) and Kyoto Encyclopedia of Genes and Genomes (KEGG) pathway enrichment analyses^54^^,^^55^^,^^56^ were conducted using the clusterProfiler R package (v4.10.0).^57^^,^^58^^,^^59^ Genes of interest—including those significantly correlated with pseudotime progression (q-value < 0.01) and DEGs identified across cell clusters or conditions (|log_2_FC| > 0.25, adjusted P < 0.05)—were converted from gene symbols to Entrez IDs using the bitr function with the org.Hs.eg.db annotation database. Functional enrichment was performed using the enrichGO and enrichKEGG functions, specifying “BP” (biological process), “MF” (molecular function), “CC” (cellular component), or “ALL” ontologies as required. Parameters were set as follows: p-value cutoff = 0.05, q-value cutoff = 0.2, and p-value adjustment method = “BH” (Benjamini-Hochberg). Background genes were defined as all expressed genes detected in the respective cell populations. For multi-group comparisons (e.g., intermediate vs. late pseudotime modules), the compareCluster function was used to compute enrichment across gene sets simultaneously, enabling comparative functional interpretation. Enrichment results were visualized using dotplot and emapplot functions, and GO terms were wrapped using stringr::str_wrap for improved readability in publication-quality figures. All visualizations were rendered using ggplot2-compatible themes to maintain consistency across the manuscript.

#### Pseudotime trajectory analysis

We performed pseudotime trajectory analysis with Monocle2 (v2.30.0)^26^ to reconstruct granulocyte lineage progression. The Seurat-integrated expression matrix was converted into a CellDataSet object using the newCellDataSet function, with cell metadata defining phenotypic annotations and gene names specifying features. After estimating size factors and dispersion parameters to normalize expression and mitigate technical noise, we ordered cells in pseudotime based on highly dispersed genes (mean expression ≥ 0.1). DifferentialGeneTest identified trajectory-associated genes, modeling Pseudotime, State, or group as covariates; genes with an FDR < 0.01 were retained for ordering. DDRTree reduced dimensionality before pseudotemporal cell ordering via orderCells. Visualizations colored cells by pseudotime, cell type, or experimental group, while heatmaps highlighted dynamic expression trends. Top-ranked genes underwent GO enrichment analysis using clusterProfiler’s enrichGO function.

#### High-dimensional weighted gene co-expression network analysis (hdWGCNA)

To identify transcriptional modules linked to disease progression, we performed high-dimensional weighted gene co-expression network analysis (hdWGCNA) separately for granulocytes and macrophages.^27^ The Seurat objects underwent initial processing with the SetupForWGCNA function, filtering genes by expression frequency (≥5% of cells). We generated metacells within each sample group using the MetacellsByGroups function to mitigate data sparsity and enhance network inference reliability. After normalizing the metacell expression matrices, we computed adjacency matrices and topological overlap matrices (TOMs). The scale-free topology criterion guided our selection of the optimal soft-thresholding power. Hierarchical clustering of TOM-based dissimilarities revealed co-expression modules, while module eigengenes (MEs) were calculated across all cells. Following module renaming and ordering by eigengene connectivity (kME), we assessed differential module eigengene (DME) expression between intermediate and late-severity samples using Student’s t-test or Wilcoxon test. Late-severity upregulated modules were cross-referenced with differentially expressed genes (DEGs) from FindMarkers, and the overlapping gene sets underwent GO enrichment analysis to characterize functional pathways. We visualized results through eigengene dot plots, hub gene rankings, dendrograms, and Venn diagrams comparing module genes with DEGs.

#### Metabolic pathway activity analysis

To assess metabolic reprogramming across disease stages, we inferred pathway-level metabolic activity with the scMetabolism R package (v0.2.1),^28^ which employs KEGG metabolic pathway gene sets to quantify single-cell pathway activity.^54^^,^^55^^,^^56^ Using the normalized expression matrix from Seurat objects, we calculated enrichment scores for each pathway in individual cells via the AUCell method, avoiding prior imputation. Macrophages and granulocytes were analyzed separately. For each cell type, we subset samples from intermediate and late-severity lung injury independently. Activity scores for over 80 KEGG metabolic pathways—including glycolysis, oxidative phosphorylation, amino acid biosynthesis, fatty acid metabolism, and glycosaminoglycan degradation—were computed using the sc.metabolism.Seurat function. Default parameters were maintained unless specified, with computations parallelized across two CPU cores. After scoring, we extracted and merged pathway-level activity matrices across stages. Group-specific mean activity scores (intermediate vs. late) were derived by averaging across cells. Pathway labels were standardized by removing suffixes through regular expressions, and redundant pathways were consolidated. Differential metabolic pathways were defined by stage-specific score differences (|Δmean score| > 0.015). These pathways were visualized in row-scaled heatmaps using the pheatmap package, with hierarchical clustering applied to rows and columns. This analysis revealed key metabolic processes enriched in late-severity macrophages and granulocytes, such as elevated oxidative phosphorylation, altered nucleotide metabolism, and modified amino acid processing, offering insights into immune cell functional adaptation during disease progression.

#### Mfuzz clustering

To investigate dynamic gene expression patterns across disease stages, we performed soft clustering using the Mfuzz package (v2.62.0).^30^^,^^31^ To extend the trajectory analysis to include a healthy baseline, we integrated a publicly available scRNA-seq dataset of healthy alveolar. Top 2,000 highly variable genes were identified from the integrated Seurat object using FindVariableFeatures, and average expression values across early, middle, and late lung injury stages were computed with AverageExpression. The resulting pseudo-bulk expression matrix was converted into an ExpressionSet object. Genes with excessive missing values (>25%) were filtered using filter.NA, and remaining missing values were imputed via the k-nearest neighbors (knn) method. Expression values were standardized using standardise, and the optimal fuzzifier (m) was estimated with mestimate. The number of clusters was set to three, corresponding to the number of time points. Clustering was conducted with mfuzz, and temporal gene expression patterns were visualized using mfuzz.plot and mfuzz.plot2, with stages ordered explicitly. Genes from selected clusters were subsequently subjected to Gene Ontology enrichment analysis to interpret their functional relevance.

#### Cell-cell communication analysis

To investigate intercellular signaling dynamics during disease progression, we employed the CellChat package (v1.6.1) for ligand-receptor interaction analysis.^32^^,^^33^ The preprocessed Seurat objects were stratified into intermediate and late-severity groups according to sample metadata, with gene expression matrices extracted for each stage. Cell type annotations curated from the dataset defined the labels for communication analysis. Normalized expression data and cell identity metadata were used to construct separate CellChat objects for each condition, with the human ligand-receptor reference database (CellChatDB.human) applied to focus on secreted signaling pathways. Overexpressed signaling genes and interactions were identified using identifyOverExpressedGenes and identifyOverExpressedInteractions, while protein-protein interaction context was integrated via projectData. Communication probabilities were calculated with computeCommunProb, excluding interactions involving fewer than 10 cells. Significant interactions were identified using a permutation test framework implemented in CellChat, retaining only ligand-receptor pairs with p < 0.05. A comprehensive list of all significant interactions is provided in Table S3. Pathway-level networks were aggregated using computeCommunProbPathway and aggregateNet, with total interaction counts and signal strength visualized through circle plots, heatmaps, and chord diagrams. Merged CellChat objects (mergeCellChat) facilitated comparison of interaction numbers and weights via compareInteractions, while netVisual_diffInteraction and interaction strength heatmaps quantified differential patterns. Signaling role centrality was assessed using netAnalysis_signalingRole_scatter and netAnalysis_signalingRole_heatmap to classify cell groups as dominant signal senders or receivers. Functional and structural network similarities between stages were computed with computeNetSimilarityPairwise, followed by manifold embedding (netEmbedding) and clustering (netClustering) to identify conserved or divergent communication modules. The netVisual_bubble function highlighted differential ligand-receptor pair usage across cell types, revealing upregulated and downregulated signaling axes. Pathway-specific information flow was ranked via rankNet, and source-target contributions were analyzed using netAnalysis_contribution. For visualization (Figure 6F), signaling pathways were prioritized based on two criteria: (i) significant differential information flow between stages, and (ii) biological relevance to ALI pathogenesis (e.g., TNF, IFN, RESISTIN) supported by prior literature. Transcriptomic validation of signaling differences was performed by directly comparing gene expression patterns between conditions with plotGeneExpression.

### Quantification and statistical analysis

Differentially expressed genes (DEGs) in scRNA-seq data were identified using the Wilcoxon rank-sum test with a threshold of adjusted p < 0.05 and |log2FC| > 0.25. For module scores and RT-qPCR data comparisons, the Wilcoxon rank-sum test was used as appropriate based on data distribution. For multi-group comparisons in animal experiments (e.g., Saline vs. Intermediate vs. Late vs. Late+p38i), one-way ANOVA with Tukey’s post-hoc test was performed. All analyses were conducted in R (v 4.3.0). Statistical significance was defined as p < 0.05. For pooled-severity scRNA-seq comparisons, statistical results were used to support hypothesis generation and were complemented by targeted experimental validation. Asterisks used in figures denote statistical significance as follows: ∗p < 0.05, ∗∗p < 0.01, ∗∗∗p < 0.001, and ∗∗p < 0.0001; ns, not significant. The statistical test used for each comparison is indicated in the corresponding figure legend.

## Supplemental information

Document S1. Figures S1–S4

Table S1. Clinical characteristics of patients with intermediate- and late-severity ALI, related to STAR Methods

Table S2. Gene sets used for functional signature scoring, related to Figure 3

Table S3. Significant ligand-receptor interactions inferred by CellChat, related to Figure 6

Table S4. Sequences of primers used for RT-qPCR validation, related to Figure 3, and STAR Methods
