# Supplementary material for: Dynamics of severity-associated immune remodeling by granulocytes and macrophages in acute lung injury
Source: iScience. 2026 Apr 21;29(5):115816. doi: 10.1016/j.isci.2026.115816 (PMC13157102; doi:10.1016/j.isci.2026.115816)
Supplement: Document S1. Figures S1–S4 [file mmc1.pdf]

iScience, Volume *29*

Supplemental information

Dynamics of severity-associated immune remodeling by granulocytes and macrophages in acute lung injury

Wanqin Zeng, Caijin Wang, Chengjian Cao, Chunlai Nie, Yingjun Fan, Yi Zhang, and Zhongshan He

Supplementary Figure

A

	Intermediate	Late
Estimated Number of Cells	5,912	5,063
Mean Reads per Cell	62,732	77,378
Median Genes per Cell	925	857
Number of Reads	370,869,535	391,766,684
Valid Barcodes	97.10%	97.30%
Sequencing Saturation	85.70%	89.90%
Q30 Bases in Barcode	95.20%	95.70%
Q30 Bases in RNA Read	94.70%	94.90%
Q30 Bases in UMI	96.70%	97.00%
Reads Mapped to Genome	96.80%	96.70%
Reads Mapped Confidently to Genome	94.30%	94.80%
Reads Mapped Confidently to Intergenic Regions	4.00%	4.20%
Reads Mapped Confidently to Intronic Regions	25.60%	26.80%
Reads Mapped Confidently to Exonic Regions	64.70%	63.80%
Reads Mapped Confidently to Transcriptome	82.90%	82.30%
Reads Mapped Antisense to Gene	6.90%	7.70%
Fraction Reads in Cells	82.30%	66.80%
Total Genes Detected	26,377	26,099
Median UMI Counts per Cell	2,141	2,473

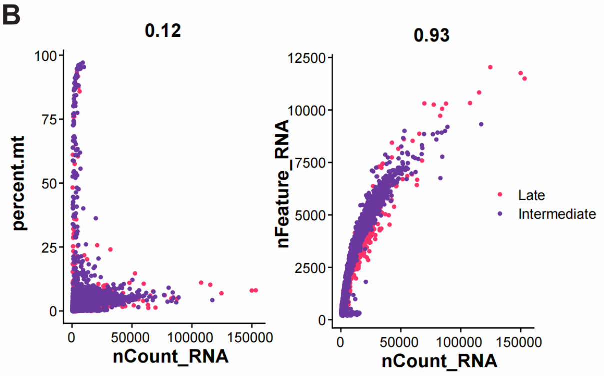

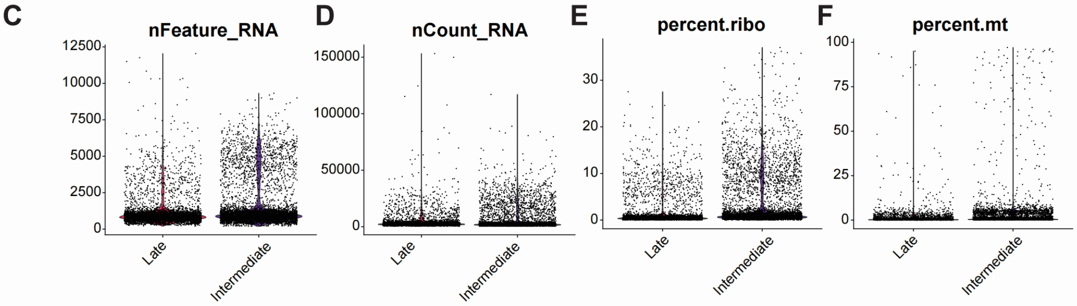

Figure S1. Quality controls for single-cell RNA-sequencing (scRNAseq) analysis of BALF samples. Related to Figure 2.

A, Summary of quality control metrics for scRNA-seq data from bronchoalveolar lavage fluid (BALF) samples of patients with intermediate-stage or late-stage acute lung injury. Metrics include the number of cells, total and mean reads per cell, gene detection, UMI counts, and percentages of reads mapped to genomic, exonic, and transcriptomic regions. The table also reports the percentages of valid barcodes, antisense reads, and Q30 base percentages for barcode, RNA read, and UMI sequences.

B, Scatterplots showing the relationship between mitochondrial gene expression percentage (percent.mt) and total UMI counts (nCount_RNA) (left) as well as the relationship between the number of detected features (nFeature_RNA) and total UMI counts (nCount_RNA) (right) for cells from intermediate-stage and late-stage lung injury samples. These plots were used to identify and exclude outlier cells during quality control.

C–F, Violin plots showing the distribution of the number of genes (nGene) (**C**), number of unique molecular identifiers (nUMI) (**D**), percentage of ribosomal gene reads (percent.ribo) (**E**), and percentage of mitochondrial gene reads (percent.mito) (**F**) for individual cells in each suspension dataset. Each data point represents the corresponding value of a single cell, with distributions highlighting variations across the intermediate-stage and late-stage datasets.

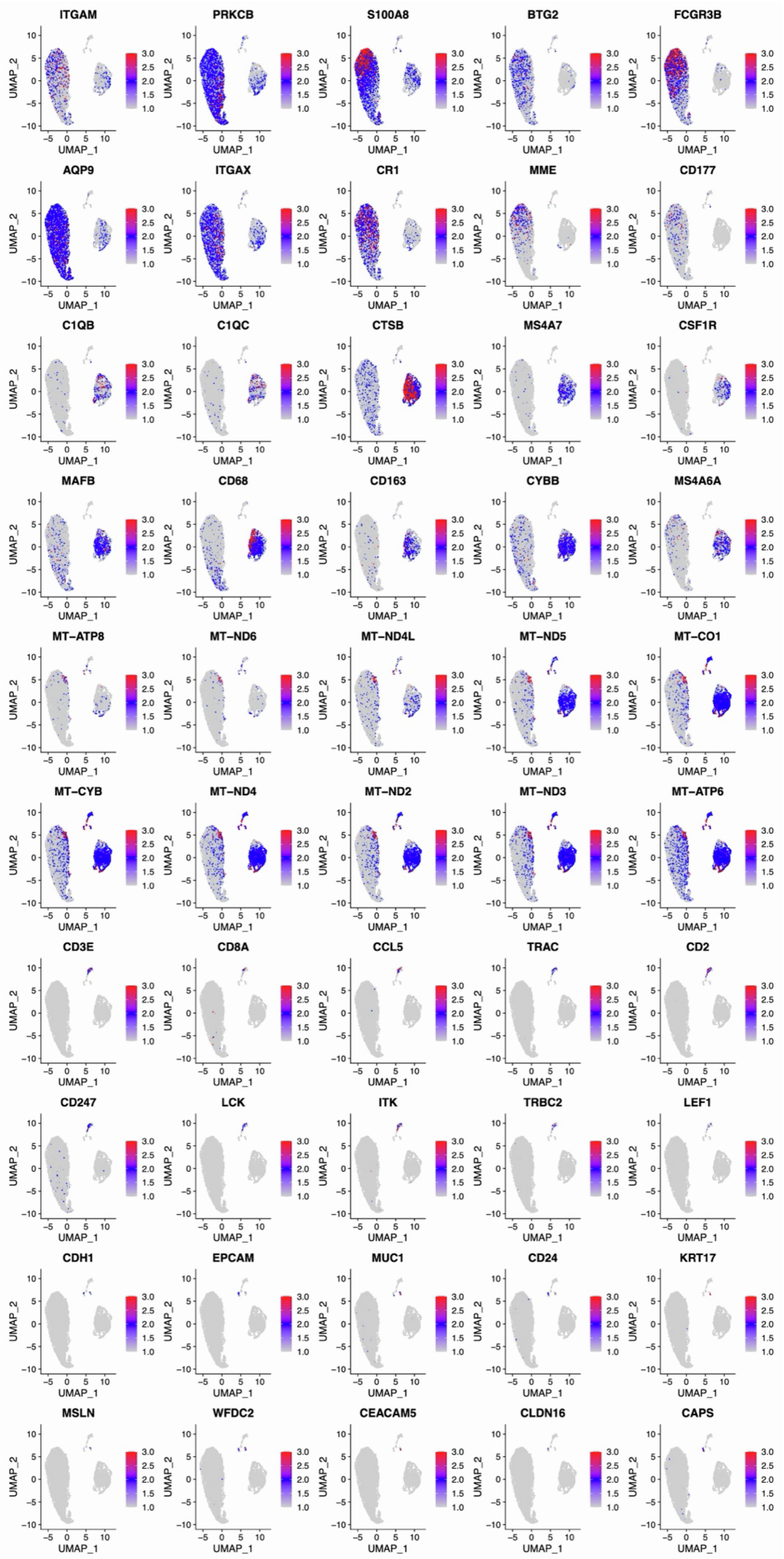
ITGAM
PRKCB
S100A8
BTG2
FCGR3B
AQP9
ITGAX
CR1
MME
CD177
C1QB
C1QC
CTSB
MS4A7
CSF1R
MAFB
CD68
CD163
CYBB
MS4A6A
MT−ATP8
MT−ND6
MT−ND4L
MT−ND5
MT−CO1
MT−CYB
MT−ND4
MT−ND2
MT−ND3
MT−ATP6
CD3E
CD8A
CCL5
TRAC
CD2
CD247
LCK
ITK
TRBC2
LEF1
CDH1
EPCAM
MUC1
CD24
KRT17
MSLN
WFDC2
CEACAM5
CLDN16
CAPS
UMAP_1
UMAP_2

Figure S2. Marker gene expression for major cell types in lung injury samples. Related to Figure 2.

Feature plots showing the expression patterns of representative marker genes used for cell type annotation in intermediate- and late-stage lung injury samples. Granulocytes were identified by high expression of S100A8, FCGR3B, and ITGAX; macrophages by CTSB, CD68, and MS4A7; epithelial cells by EPCAM, KRT17, and CDH1; lymphocytes by CD3E, TRAC, and LCK; and a metabolically active MT_hi population by MT-ND4L, MT-CO1, and MT-CYB. Each panel displays the UMAP projection of all cells, with the color scale indicating relative gene expression levels (log-normalized). These canonical markers facilitated robust identification of major cellular compartments in the lung immune microenvironment.

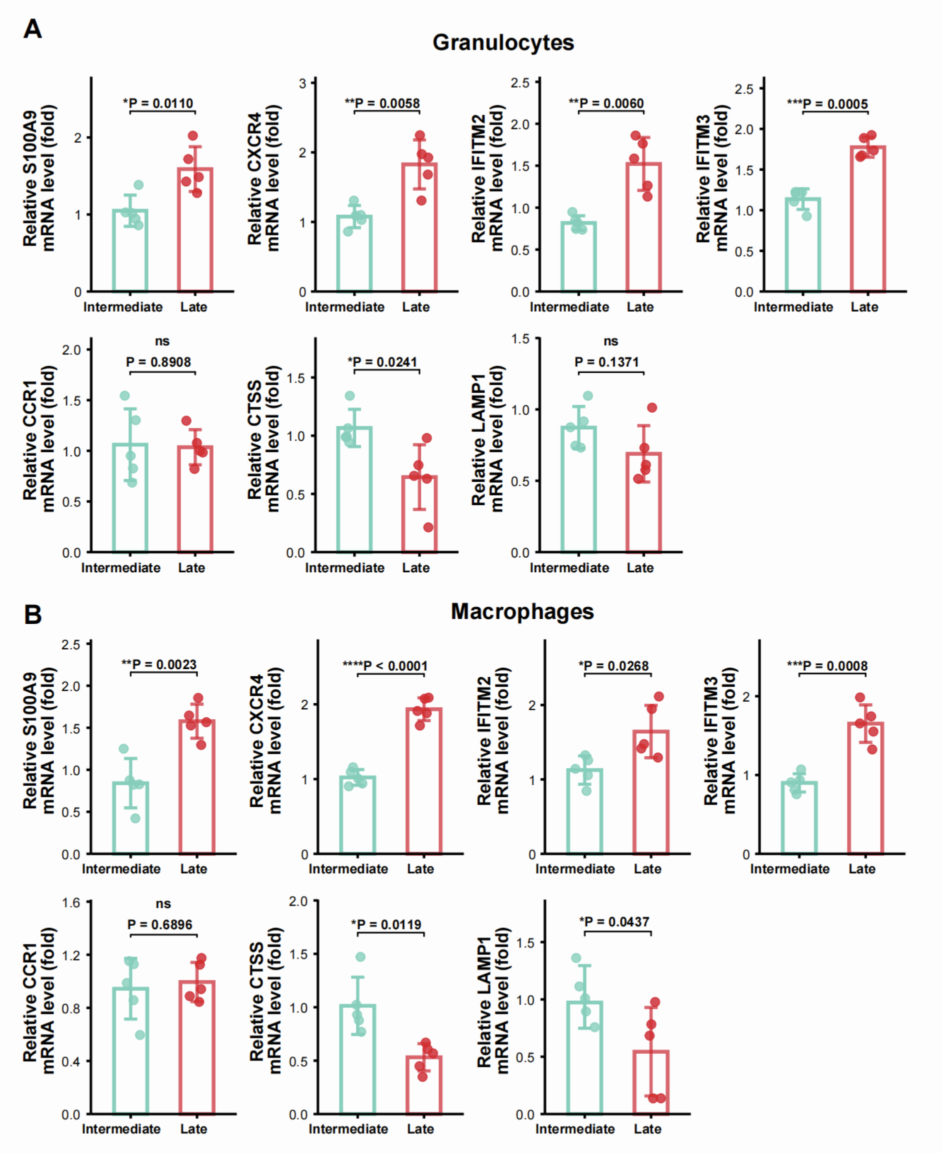

Figure S3. Independent RT-qPCR validation of stage-specific transcriptional reprogramming in primary human BALF granulocytes and macrophages. Related to Figure 3.

(**A**-**B**) Relative mRNA expression of key inflammatory (S100A9, CXCR4, IFITM2, IFITM3, CCR1) and lysosomal (CTSS, LAMP1) genes evaluated by RT-qPCR in FACS-sorted primary human BALF granulocytes (**A**) and macrophages (**B**). Cells were isolated from an independent validation cohort of patients with intermediate- (*n* = 5) and late-stage (*n* = 5) ALI (distinct from those used for scRNA-seq). Data were normalized to the housekeeping gene GAPDH and are presented as fold change relative to the intermediate-

stage group. Consistent with the scRNA-seq findings, these independent validation data confirm that late-stage cells exhibit significant upregulation of inflammatory hubs and downregulation of lysosomal markers. Data were analyzed using an unpaired two-tailed Student's t-test and shown as mean ± S.D. Statistical significance is indicated as *P < 0.05, **P < 0.01, ***P < 0.001, and ****P < 0.0001; ns, not significant.

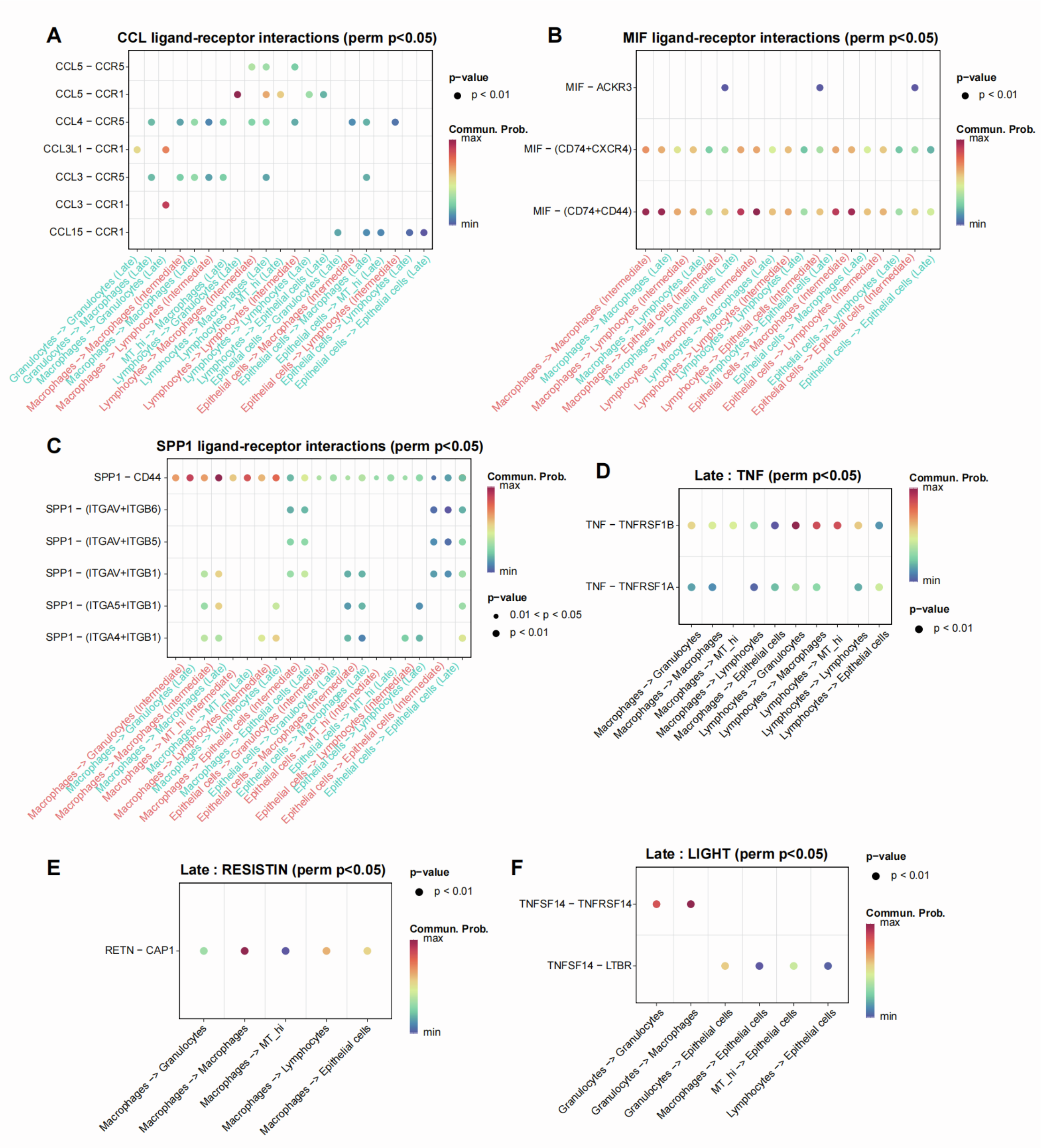

Figure S4. Ligand–receptor interactions underlying stage-dependent remodeling of intercellular communication. Related to Figure 6.

(A–C) Bubble plots showing differential ligand–receptor interactions (permutation p < 0.05) for CCL (A), MIF (B), and SPP1 (C) signaling across major sender–receiver cell compartments in intermediate and late-stage lung injury samples (CellChat merged object). Dot color indicates inferred communication probability (higher = stronger), and dot size encodes permutation-based significance (larger dots indicate smaller p-values, e.g., p < 0.01).

(D–F) Bubble plots for late-stage–specific significant interactions (permutation $p < 0.05$) in TNF (D), RESISTIN/RETN (E), and LIGHT/TNFSF14 (F) signaling, highlighting pathways that did not reach significance in the intermediate stage under the same permutation framework but emerged as significant in late-stage samples.

All results were inferred by CellChat; only ligand–receptor pairs passing the permutation threshold are displayed.
